# Multi-omics integration and *in vitro* validation identify IL4R, IMPA2, and PRR4 as key therapeutic targets in chronic rhinosinusitis with nasal polyps

**DOI:** 10.3389/fimmu.2026.1792878

**Published:** 2026-06-15

**Authors:** Bo Wei, Weigang Gan, Jiaxin Li, Feng Wang, Jun Wang, Binbin Wang, Jiaoyu He, Feng Liu

**Affiliations:** 1 Department of Otolaryngology Head and Neck Surgery, West China Hospital, Sichuan University, Chengdu, China; 2 Renji Medical Research Center, Chengdu Second People’s Hospital, Chengdu, China

**Keywords:** chronic rhinosinusitis with nasal polyps, immune infiltration, machine learning, Mendelian randomization, molecular docking, single-cell sequencing

## Abstract

**Background:**

Chronic rhinosinusitis with nasal polyps (CRSwNP) is a common inflammatory disease with complex pathogenesis. This study aims to screen out key molecular markers and potential therapeutic targets through multi-omics data integration.

**Methods:**

Single-cell RNA sequencing data (GSE276503) and transcriptome data (GSE136825, GSE179265) from the GEO database were integrated. Quality control, normalization, clustering, annotation, multi-omics Integration and in Vitro Validation were performed.

**Results:**

4460 differentially expressed genes and 732 hub genes were identified. MR yielded 1673 disease-related genes. After integrating with druggable genes, 43 candidate genes were screened, and they were enriched in complement/coagulation cascades and hematopoietic cell lineage pathways. Machine learning identified three key genes: IL4R and IMPA2 (upregulated in CRSwNP) and PRR4 (downregulated). Immune analysis showed increased monocytes, M2 macrophages, and neutrophils, with decreased memory CD4 T cells in CRSwNP. We constructed a ceRNA network around the key genes and identified transcription factors including GATA2. Drug prediction yielded 26 potential drugs, with molecular docking confirming strong binding of raloxifene (IL4R), luteolin (IMPA2), and metronidazole (PRR4). MR preliminarily suggested IL4R and IMPA2 as potential risk factors and PRR4 as a potential protective factor for CRSwNP. The co-location analysis further evaluated the association between genetic variation of key genes and CRSwNP. Knockdown of IL4R or IMPA2, as well as overexpression of PRR4, significantly attenuated lipopolysaccharide (LPS)-induced cellular injury by reducing apoptosis, suppressing inflammatory responses, and restoring epithelial barrier integrity (all P < 0.001). These findings confirmed the protective effects of targeting these key genes against CRSwNP-related inflammation and epithelial dysfunction.

**Conclusion:**

This multi-omics approach identified three key genes in CRSwNP pathogenesis and their regulatory mechanisms. *In vitro* functional experiments further validated that modulation of these key genes can effectively protect nasal epithelial cells from inflammatory injury, providing new molecular targets and potential therapeutic drugs for CRSwNP diagnosis and treatment.

## 1 Introduction

Chronic rhinosinusitis with nasal polyps (CRSwNP) represents a prevalent inflammatory condition affecting the sinonasal mucosa, distinguished by prolonged inflammatory processes and polyp development within nasal cavities (1). This disorder impacts approximately 1–7% of individuals globally, with patients frequently experiencing severe fatigue, mood disturbances, and cognitive impairments that substantially diminish their daily functioning (2). The pathogenesis of CRSwNP is extremely complex and not fully understood (3). From a histological perspective, CRSwNP typically presents with a Th2-type inflammatory response, characterized by infiltration of eosinophils and increased expression of Th2 cytokines such as IL-4, IL-5, and IL-13 (4, 5). In addition, epithelial barrier dysfunction, autoimmune responses, and dysbiosis of the microbiome also play significant roles in the disease pathogenesis (6–8). Currently, the diagnosis of CRSwNP primarily relies on symptom assessment, nasal endoscopy, and imaging examinations. However, the symptoms lack specificity, nasal endoscopy requires specialized equipment, and imaging cannot effectively distinguish between different inflammatory subtypes (9, 10). Moreover, laboratory tests such as serum IgE levels and histological analysis provide limited information with insufficient specificity and sensitivity (11). Therefore, the lack of specific biomarkers that can early and accurately identify CRSwNP remains a major challenge in clinical practice.

Treatment for CRSwNP includes both medical and surgical management (12). For patients unresponsive to medical treatment, endoscopic sinus surgery is the standard approach, but the postoperative recurrence rate is as high as 35-40% (13). In recent years, biologics such as anti-IgE, anti-IL-4/IL-13, and anti-IL-5 antibodies have shown promising efficacy in patients with severe, refractory disease (14). However, current treatments still face challenges such as limited efficacy and a lack of individualized strategies. Improved diagnosis and treatment of CRSwNP is dependent on a deeper understanding of the molecular mechanisms underlying the condition. This is due to the significant clinical implications of such understanding for the identification of new therapeutic targets and the development of personalised treatment strategies.

Expression Quantitative Trait Loci (eQTL) and Genome-Wide Association Studies (GWAS) provide important tools for understanding the genetic basis of CRSwNP. eQTL analysis reveals how genetic variations regulate gene expression, while GWAS identifies genetic loci associated with disease risk (15, 16). Mendelian randomization (MR) is a way to work out the true cause and effect relationship between what causes a disease and who gets it. It does this by using information about people’s genes. This is better than other studies that look at what happens after a disease has already appeared, because it can avoid problems that make it hard to work out what the cause is (17). MR combined with eQTL and GWAS data has made significant breakthroughs in various diseases, including the identification of causative genes, evaluation of drug target efficacy, and elucidation of molecular disease subtypes (18–20). Compared to traditional methods, multi-omics integration analysis can help us to understand molecular mechanisms, improve the accuracy of candidate gene selection, and reveal gene interaction networks. Given the complexity of the pathogenesis of CRSwNP and its overlapping with multiple comorbidities, traditional statistical correlation analysis is highly susceptible to interference from confounding factors and reverse causality; MR uses genetic variations as a control variable to sever the aforementioned confounding at the genetic level, thereby still providing more reliable causal inferences in the context of complex inflammation. Furthermore, independent transcriptome analysis cannot distinguish causal relationships from correlations, while GWAS provides genetic associations but lacks functional information, and even eQTL data alone cannot establish the causal direction; therefore, the systematic integration of these three methods can identify candidate genes while inferring their causal nature, thereby generating mechanistic insights that surpass any single method. However, there is still a lack of systematic multi-omics integration studies in the CRSwNP field, and the functional significance and causal relationships of genetic variations and differentially expressed genes are not well understood.

This study aims to systematically analyze the molecular pathogenesis of CRSwNP by integrating single-cell RNA sequencing data, transcriptomic data, GWAS data, and eQTL data. We will use a series of bioinformatics methods to identify key genes, evaluate causal relationships between gene expression and disease risk through MR, construct competing endogenous RNA (ceRNA) and transcription factor (TF) regulatory networks to uncover multi-level regulatory mechanisms, and predict potential therapeutic drugs. This multi-omics integration strategy is expected to identify key molecular biomarkers and potential therapeutic targets for CRSwNP, providing a new theoretical foundation for early diagnosis and precision treatment.

## 2 Methods

### 2.1 Data sources

Transcriptome datasets GSE136825, GSE179265, and single-cell dataset GSE276503 related to CRSwNP were downloaded from the GEO database (https://www.ncbi.nlm.nih.gov/geoprofiles/). GSE136825, based on the GPL20301 platform, contained 42 CRSwNP and 28 normal tissue samples and was used as the training set; GSE179265, based on the GPL24676 platform, contained 17 CRSwNP and 7 normal tissue samples and was used as the validation set. The single-cell sequencing dataset GSE276503 (platform: GPL29480) included 15 CRSwNP and 2 control samples.

CRSwNP data was downloaded from the IEU OpenGWAS database (https://gwas.mrcieu.ac.uk/) with the data ID ukb-a-541, including 1637 chronic rhinosinusitis cases and 335562 control samples.

The eQTLs data was obtained from the eQTLGen consortium database (https://eqtlgen.org/), containing 15695 genes. Finally, 5631 druggable genes were retrieved from the DGIdb database (https://dgidb.org/) (**Supplementary Table 1**).

### 2.2 Single-cell analysis

Seurat (v4.0.1) was used for quality control (nFeature RNA: 200-5000, percent.mt < 5%), normalization (LogNormalize), scaling, and PCA-based dimensionality reduction. Cell clustering and annotation were performed using FindClusters and SingleR. UMAP visualization and cell proportion analysis were conducted. CellChat was used for cell-cell interaction analysis.

### 2.3 Differential expression analysis

DEGs were identified using the limma package with thresholds of |FC| > 1.5 and nominal P < 0.05 (unadjusted for multiple testing) in GSE136825. The fold-change threshold of 1.5 was selected to retain genes with biologically meaningful expression differences while controlling the false-positive rate to a practical level; this approach is widely adopted in transcriptomic screening studies and was applied here as an initial candidate generation step, with subsequent multi-step validation serving to filter spurious findings.

### 2.4 Candidate gene acquisition

WGCNA identified co-expressed modules and hub genes (|GS| > 0.5, |MM| > 0.1) in CRSwNP-associated modules (P < 0.05). Notably, the relatively lenient |MM| > 0.1 threshold was intentionally adopted as an initial broad-screening strategy to avoid premature exclusion of potentially important candidate genes at this early stage. MR analysis using TwoSampleMR identified causal genes with strict quality control (P < 5×10^-8^, F ≥ 10, LD r² < 0.1). Candidate genes were obtained by intersecting DEGs, MR-identified genes, hub genes, and druggable genes.

### 2.5 Enrichment analysis

GO and KEGG enrichment analyses were performed using DAVID (P < 0.05, unadjusted for multiple comparisons). GSEA explored pathway differences between CRSwNP and controls. Given the exploratory nature of this enrichment screening and the multi-dimensional downstream validation strategy employed in this study, nominal P values were used as the significance criterion; results should be interpreted as hypothesis-generating rather than definitive.

### 2.6 Machine learning and risk prediction

Three machine learning algorithms were applied to identify key predictive genes from the intersection of differentially expressed genes.

Random Forest (rfPermute): A random forest model was constructed using the rfPermute R package with the number of decision trees set to ntree = 1, 000 and permutation test replicates set to nrep = 299. The random seed was fixed at set.seed(123) to ensure reproducibility. Feature selection was based on permutation-based importance (%IncMSE), retaining only features with a statistically significant contribution to model accuracy (permutation test P < 0.05).

LASSO Regression (glmnet): Least Absolute Shrinkage and Selection Operator (LASSO) regression was performed using the glmnet R package with a binomial distribution family (family = “binomial”) and elastic net mixing parameter α = 1 (pure LASSO penalty). The optimal regularization parameter λ (lambda.min) was selected via 10-fold cross-validation (nfold = 10) using classification error as the selection criterion (type.measure = “class”). Only features with non-zero coefficients at lambda.min were retained.

SVM Recursive Feature Elimination (mSVM-RFE): Support Vector Machine-based Recursive Feature Elimination was implemented using a linear kernel function (kernel = “linear”) with SVM penalty parameter cost = 10. An outer 10-fold cross-validation (nfold = 10) was applied, and within each fold, k = 10 sub-samplings were performed to obtain multiple weight vectors (i.e., the mSVM-RFE strategy). During hyperparameter optimization, gamma was searched over the range 2^-12^ to 2^0^, and cost was searched over the range 2^-6^ to 2^6^; the optimal feature count was determined as the number corresponding to the minimum cross-validation error. The random seed was fixed at set.seed (123).

The intersection of genes identified by all three algorithms was used to construct a nomogram-based risk prediction model via logistic regression. Model performance and clinical utility were evaluated by decision curve analysis (DCA).

### 2.7 Immune infiltration analysis

CIBERSORTx quantified 22 immune cell types. Wilcoxon rank-sum test compared infiltration differences, and Pearson correlation assessed relationships between key genes and immune cells.

### 2.8 Regulatory network analysis

PPI networks were constructed using STRING and visualized with Cytoscape. miRNet predicted miRNAs and lncRNAs to construct ceRNA networks. Transcription factors were predicted using JASPAR database.

### 2.9 Drug prediction and molecular docking

Potential drugs were predicted using Enrichr (DSigDB) and CMAP databases. AutoDock Vina performed molecular docking to calculate binding energies and analyze interaction modes.

### 2.10 Expression validation

Key gene expression was validated in training and validation sets using Wilcoxon tests. ROC curves evaluated diagnostic performance (AUC). UMAP visualization showed expression patterns in single-cell data.

### 2.11 MR validation and colocalization

Two-sample MR (TwoSampleMR package) validated causal relationships using IVW method and sensitivity analyses (MR-Egger, weighted median, etc.). Colocalization analysis (coloc package) assessed shared causal variants using Bayesian methods (prior probabilities: 1×10^-4^ for trait-specific, 1×10^-5^ for shared variants). Results were visualized using LocusCompareR.

### 2.12 Experimental validation

#### 2.12.1 Experiment design, cell culture and lipopolysaccharide -induced CRSwNP model

The nasal epithelial cells (HNEpC; cat. CP-H252, Procell) were cultured in RPMI-1640 supplemented with 10% fetal bovine serum and 1% penicillin-streptomycin at 37 °C in a humidified atmosphere containing 5% CO_2_. To establish the *in vitro* CRSwNP model, HNEpC were treated with lipopolysaccharide (LPS, Sigma-Aldrich) at various concentrations (0.125, 0.25, 0.5, 1, 2, 4 μg/mL) for 6 hours. Cell viability was assessed using the Cell Counting Kit-8 (CCK-8, Dojindo, CK04) according to the manufacturer’s instructions, and absorbance was measured at 450 nm using a microplate reader (Thermo Fisher Scientific, Multiskan FC).

#### 2.12.2 Experimental groups and gene manipulation

The experimental groups were designed as follows: Control group (untreated HNEpC), LPS group (HNEpC treated with optimal LPS concentration), LPS+si-IL4R group (LPS treatment combined with IL4R siRNA transfection), LPS+si-IMPA2 group (LPS treatment combined with IMPA2 siRNA transfection), LPS+oe-PRR4 group (LPS treatment combined with PRR4 overexpression plasmid transfection), and LPS+NC group (LPS treatment combined with negative control vector transfection). Gene-specific siRNAs targeting IL4R, IMPA2, and CCN4, along with PRR4 and CCN4 overexpression plasmids and corresponding negative controls were commercially obtained. For siRNA-mediated knockdown experiments, HNEpC were transfected with siRNA at a final concentration of 50–100 nM using Lipofectamine 3000 (Thermo Fisher Scientific) for 24 hours, followed by LPS treatment for 6 hours. For overexpression experiments, cells were transfected with 2 μg plasmid DNA per well using Lipofectamine 3000 for 48 hours, followed by LPS treatment for 6 hours. Transfection efficiency (≥70%) was verified by quantitative real-time PCR (qRT-PCR) and Western blot analysis prior to subsequent experiments.

#### 2.12.3 RNA extraction and quantitative real-time PCR

Total RNA was extracted from tissue specimens or cultured cells using RNAiso Easy reagent (Takara, TCH020) according to the manufacturer’s protocol. RNA concentration and purity were assessed using a NanoDrop 2000 spectrophotometer (Thermo Fisher Scientific). First-strand cDNA was synthesized from 1 μg total RNA using the PrimeScript™ RT Reagent Kit (Takara, RR047A). qRT-PCR was performed using TB Green^®^ Premix Ex Taq™ (Takara, RR420A) on a Gentier 96 Real-Time PCR System (Tianlong). The thermal cycling conditions consisted of initial denaturation at 95 °C for 30 seconds, followed by 40 cycles of 95 °C for 5 seconds and 60 °C for 30 seconds. Melting curve analysis was performed from 65 °C to 95 °C. The primer sequences are listed in **Supplementary Table 2**. Relative gene expression was calculated using the 2^-ΔΔCt^ method with GAPDH as the reference gene.

#### 2.12.4 Western blot analysis

Cells or tissues were lysed in RIPA buffer (Beyotime, P0013C) supplemented with protease and phosphatase inhibitor cocktail (Beyotime, P1045) on ice for 30 minutes. Protein concentrations were determined using the BCA Protein Assay Kit (Epizyme Biotech, ZJ101L). Equal amounts of protein (30 μg) were separated by 4-20% SDS-PAGE (Servicebio, G2037) and transferred onto 0.45-μm PVDF membranes (Millipore, IPVH00010). Membranes were blocked with 5% non-fat milk in TBST for 1 hour at room temperature, then incubated overnight at 4 °C with primary antibodies against ZO-1 (1:1000, ABclonal, A0659), Claudin-1 (1:1000, Proteintech, 28674-1-AP), Occludin (1:1000, ABclonal, A24601), and GAPDH (1:1000, Abcam, ab8245). After washing three times with TBST, membranes were incubated with HRP-conjugated goat anti-rabbit IgG (1:5000, Abcam, ab205718) for 1 hour at room temperature. Protein bands were visualized using ECL chemiluminescence reagent (Tanon, 180-5001) and detected with a Tanon 5200 imaging system. Band intensities were quantified using ImageJ software (version 1.52r, NIH).

#### 2.12.5 Enzyme-linked immunosorbent assay

Cell culture supernatants were collected at 24 and 48 hours post-treatment. The concentrations of inflammatory cytokines (IL-1β, TNF-α and TSLP) were measured using commercially available ELISA kits according to the manufacturers’ instructions. Absorbance was measured at the appropriate wavelength using a microplate reader (Thermo Fisher Scientific, Multiskan FC).

#### 2.12.6 Cell function detection

HNEpC were seeded in 96-well plates at a density of 5×10^3^ cells per well. After treatment, 10 μL of CCK-8 solution (Dojindo, CK04) was added to each well and incubated for 4 hours at 37 °C. Absorbance at 450 nm was measured using a microplate reader. Cells were stained with Annexin V-FITC and propidium iodide (PI) using the Annexin V-FITC Apoptosis Detection Kit (Beyotime, C1062S) according to the manufacturer’s protocol. Apoptotic cells were analyzed by flow cytometry (BD Accuri C6 Plus).

### 2.13 Statistical analysis

All experiments were performed in triplicate and repeated at least three times independently. Data are presented as mean ± standard deviation (SD). Statistical comparisons between two groups were performed using Student’s t-test, while comparisons among multiple groups were analyzed using one-way ANOVA followed by Tukey’s or LSD *post hoc* test. Statistical analyses were conducted using SPSS 25.0 and GraphPad Prism version 10.1.2. P < 0.05 was considered statistically significant.

R programming language (v 4.3.2) was used for bioinformatics analysis. Mendelian randomization analysis results were expressed as odds ratios (OR) representing the impact of exposure factors on outcome occurrence.

## 3 Results

### 3.1 Single-cell analysis to explore cellular heterogeneity

**Figure 1:**
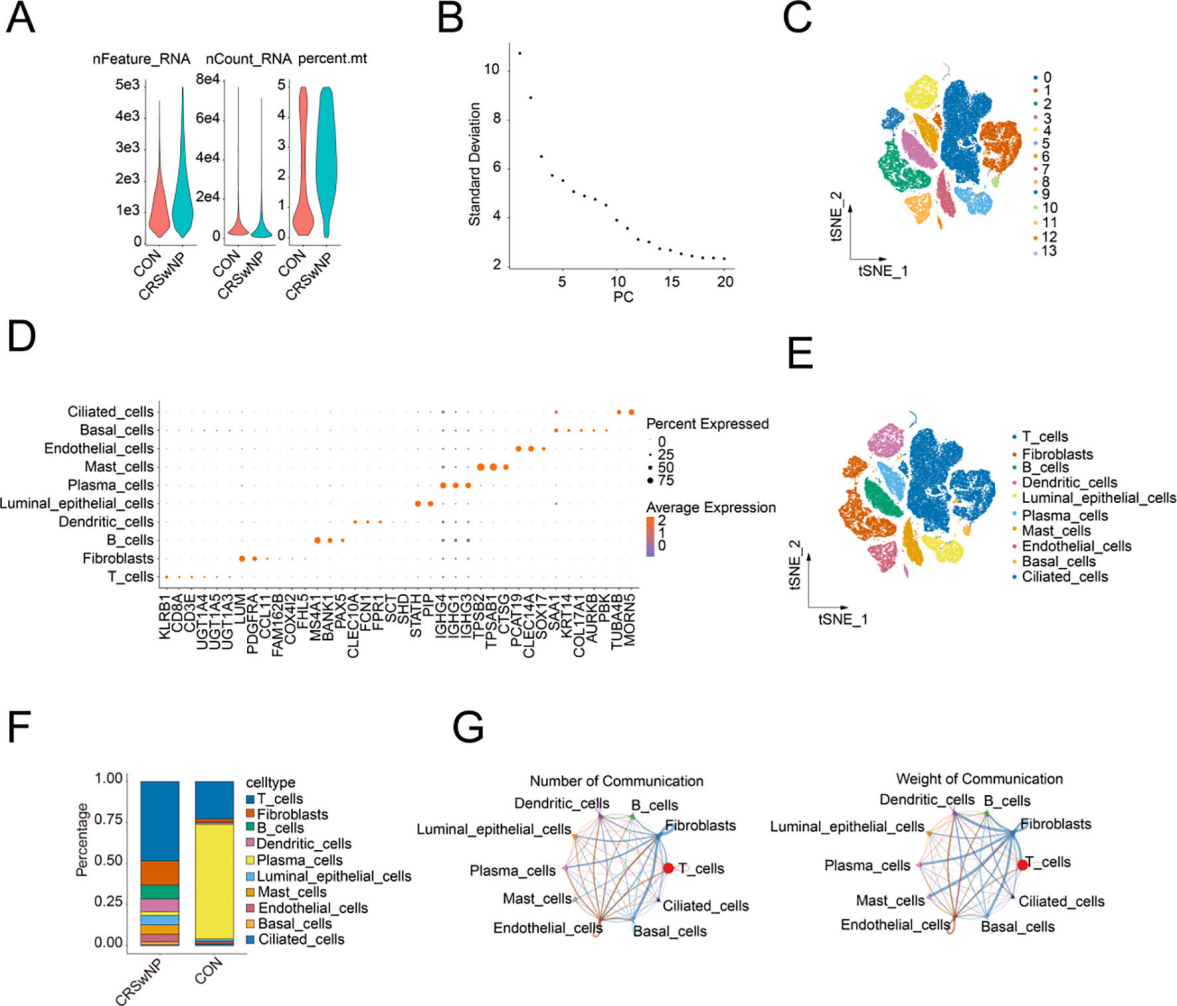
Cell composition and intercellular communication network characteristics revealed by single-cell transcriptomics analysis. **(A)** Single-cell data quality control results. **(B)** Scree plot showing the standard deviation explained by each principal component (PC). **(C)** tSNE visualization showing cell clusters. **(D)** Dot plot displaying gene expression patterns of different cell types. **(E)** tSNE visualization of annotation results. **(F)** Stacked bar chart comparing cell composition proportions between CRSwNP and control groups. **(G)** Intercellular communication network diagram, showing communication numbers and communication strength.

In this study, single-cell analysis was performed and obtained high-quality core cells for subsequent analysis (**Figure 1A**). The 17 samples in the single-cell dataset were combined and subjected to PCA, with the top 20 PCs (P < 0.05) ultimately identified (**Figure 1B**). Then, tSNE and UMAP algorithms were used to classify the core units into 14 distinct cell clusters (**Figure 1C**). After reviewing the literature and databases, cells were annotated by matching marker genes, resulting in annotations of T cells, fibroblasts, B cells, dendritic cells, luminal epithelial cells, plasma cells, mast cells, endothelial cells, basal cells, and ciliated cells (**Figures 1D, E**). Further analysis of the distribution of different cell types in CRSwNP and CON revealed increased proportions of T cells and fibroblasts in the CRSwNP group (**Figure 1F**). To further explore whether T cells and fibroblasts play important roles in the CRSwNP process, we used the CellChat tool to analyze the number and strength of intercellular communication networks. The results showed that compared to T cells, Fibroblasts had close interactions with B cells, dendritic cells, plasma cells, mast cells, and other cell types (**Figure 1G**). In summary, fibroblasts play an important role in CRSwNP.

### 3.2 Differential expression analysis

A total of 4460 genes were significantly differentially expressed in the CRSwNP group, including 3167 upregulated genes and 1293 downregulated genes (**Supplementary Figures 1A, B**).

### 3.3 WGCNA

**Figure 2:**
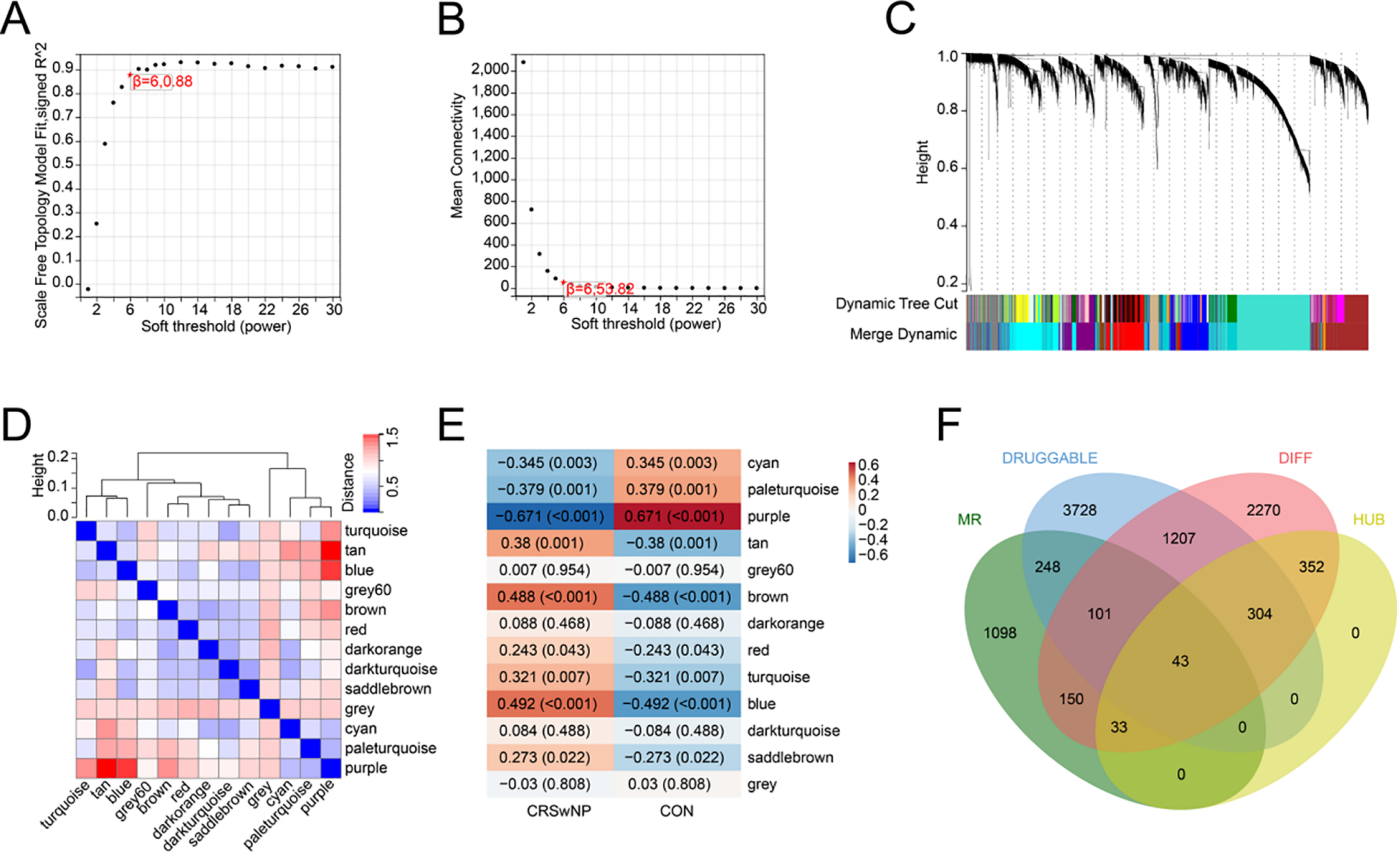
WCGNA. **(A)** Sample scale independence plot; **(B)** Sample mean connectivity plot; **(C)** Gene module clustering diagram; **(D)** Module eigenvector clustering diagram; **(E)** Module-trait relationship heatmap; **(F)** Venn diagram of DEGs, genes selected by MR, druggable genes, and hub genes from WGCNA.

The pickSoftThreshold function was used to test different soft thresholds β, and β=6 was selected to construct the adjacency matrix (**Figures 2A, B**). Based on hierarchical clustering and dynamic tree cutting algorithms, with a minimum module gene number of 30, deep split of 3, and maximum module distance of 0.25, a total of 13 gene modules were generated: cyan, paleturquoise, purple, tan, grey60, brown, darkorange, red, turquoise, blue, darkturquoise, saddlebrown, and gray (**Figure 2C**). These modules demonstrated good independence (**Figure 2D**). Among them, 9 modules were screened out: cyan, paleturquoise, purple, tan, brown, red, turquoise, blue, and saddlebrown (**Figure 2E**), from which 732 hub genes were extracted and identified. By comparing DEGs, genes selected by MR, druggable genes, and hub genes, 43 candidate genes were identified (**Figure 2F**).

### 3.4 Functional enrichment analysis of candidate genes

**Figure 3:**
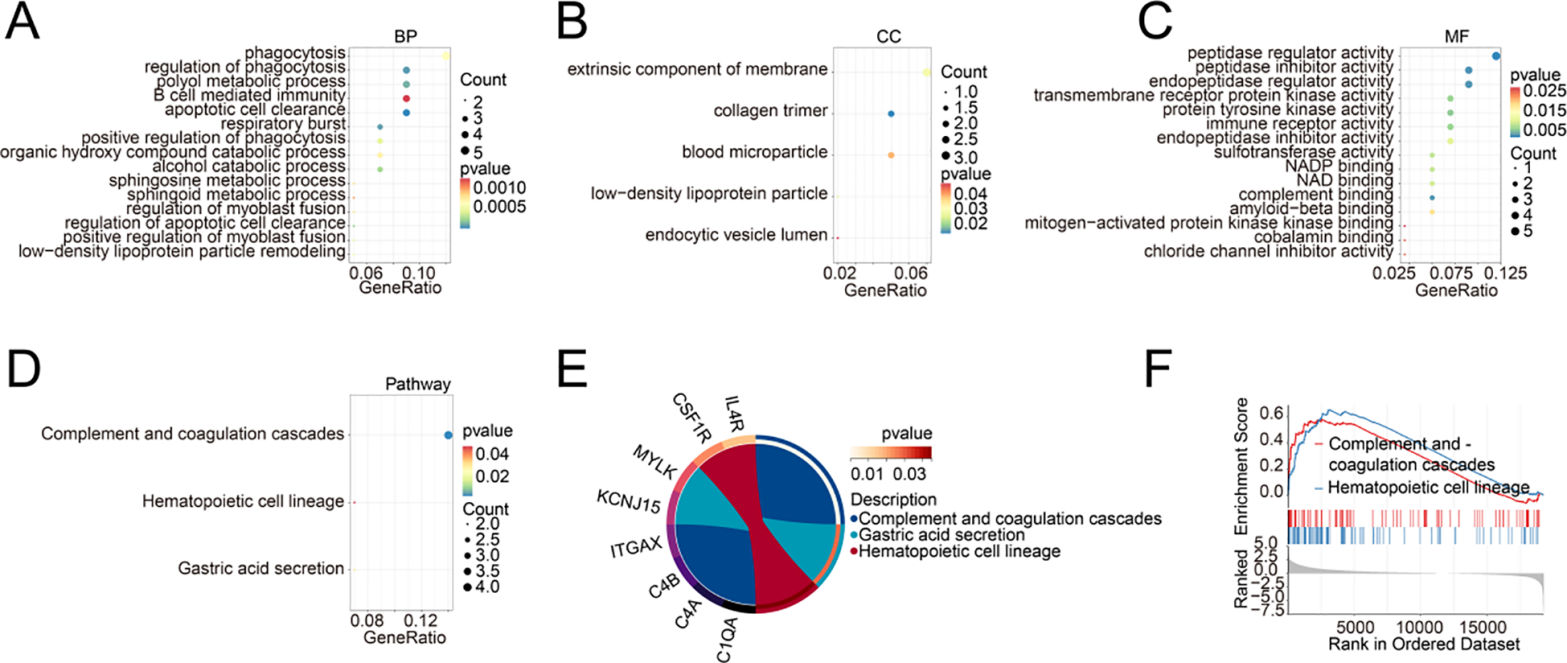
Functional enrichment analysis. **(A–C)** Bubble plots of candidate genes enriched in BP **(A)**, CC **(B)**, MF **(C)** GO terms (top 15 terms by significance); **(D)** Bubble plot of candidate genes enriched in KEGG pathways; **(E)** Connection relationships between co-expressed genes and enriched KEGG pathways; **(F)** GSEA identification of signaling pathways with significant differential expression between CRSwNP tissue samples and control tissue samples.

In GO enrichment analysis, these genes were mainly enriched in biological processes (BP) such as phagocytosis, B cell mediated immunity, apoptotic cell clearance, and polyol metabolic process (**Figure 3A**); cellular components (CC) including collagen trimer, low-density lipoprotein particle, extrinsic component of membrane, blood microparticle, and endocytic vesicle lumen (**Figure 3B**); and molecular functions (MF) such as peptidase regulator activity, endopeptidase regulator activity, complement binding, protein tyrosine kinase activity, transmembrane receptor protein kinase activity, and NADP binding (**Figure 3C**). KEGG enrichment analysis identified these pathways: complement and coagulation cascades, gastric acid secretion, and hematopoietic cell lineage (**Figures 3D, E**). GSEA analysis further confirmed that signaling pathways such as complement and coagulation cascades and hematopoietic cell lineage showed significant differences between the two groups (**Figure 3F**).

### 3.5 Machine learning screening of key genes

**Figure 4:**
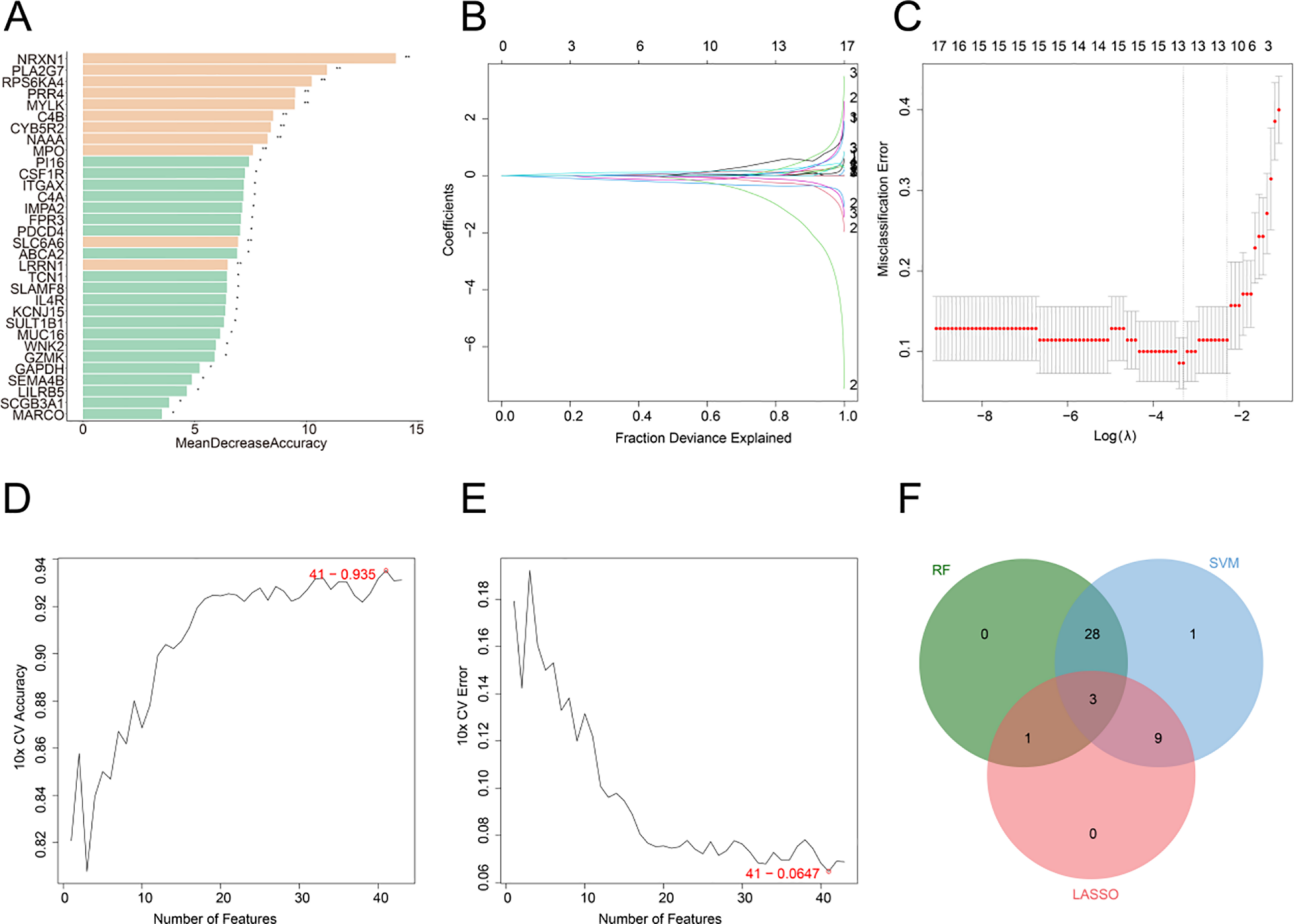
Machine learning screening of key genes. **(A)** Significant genes screened by Random Forest; **(B)** Coefficient path plot of Lasso regression model; **(C)** Binomial deviance of Lasso regression model; **(D, E)** Changes in prediction accuracy **(D)** and error values **(E)** of SVM-RFE; **(F)** Intersection of genes screened by LASSO, SVM-RFE, and Random Forest.

Machine learning methods were used to screen 43 genes in the training set, with the RF algorithm identifying 32 feature genes (**Figure 4A**). LASSO logistic regression analysis yielded 13 feature genes (**Figures 4B, C**). The SVM-RFE algorithm screened 41 feature genes (**Figures 4D, E**). Finally, the intersection of genes screened by these three algorithms yielded 3 key genes: IL4R, IMPA2, and PRR4 (**Figure 4F**). The Nomogram risk prediction model showed that high expression of IL4R and IMPA2 are risk factors for CRSwNP, while low expression of PRR4 is a risk factor for CRSwNP (**Supplementary Figure 2A**). The calibration curve indicated high predictive accuracy of the model (**Supplementary Figure 2B**). Additionally, the nomogram risk prediction model’s potential value for clinical decision-making was confirmed by the decision curve (**Supplementary Figure 2C**).

### 3.7 Immune infiltration analysis

**Figure 5:**
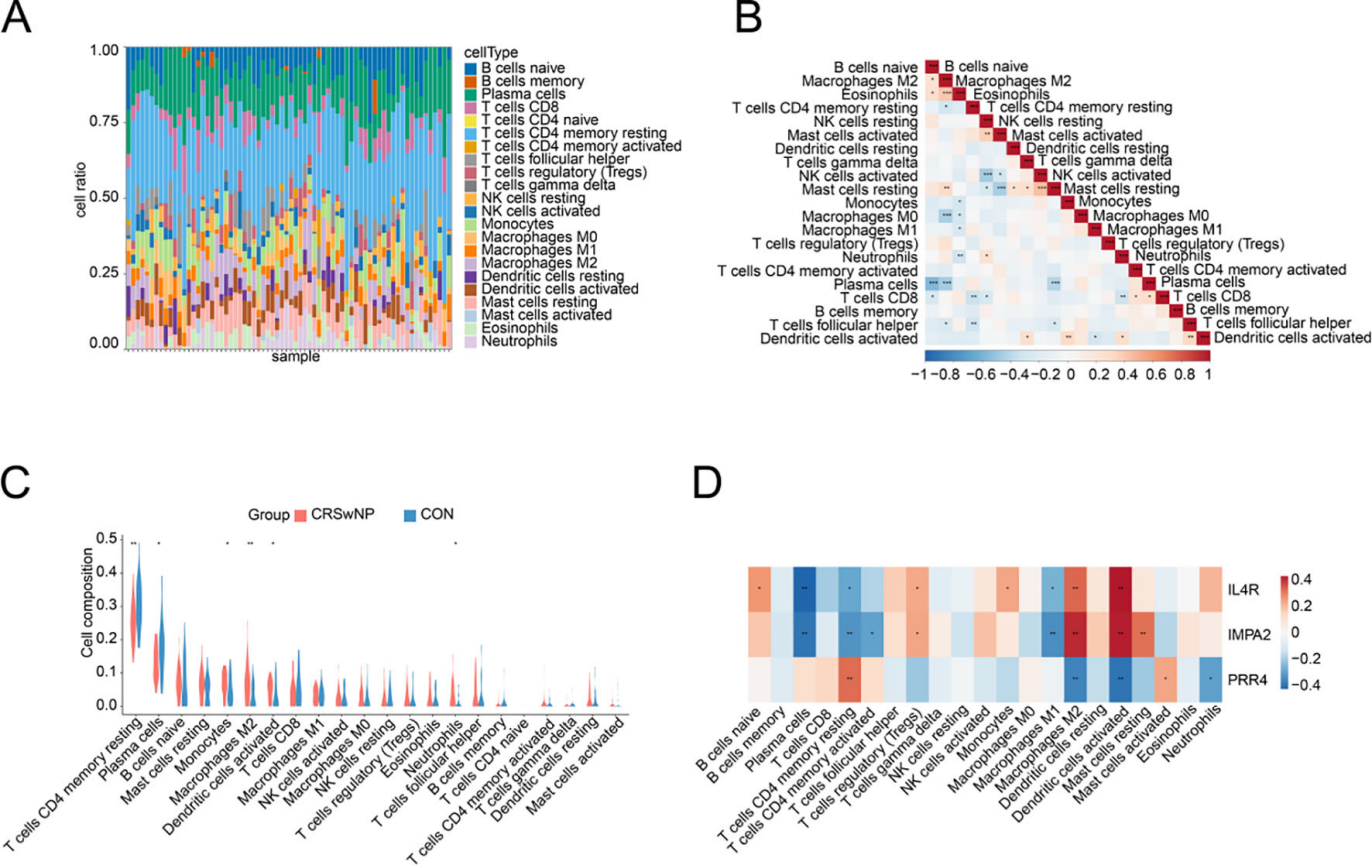
Immune infiltration analysis. **(A)** Immune cell infiltration proportions in all samples; **(B)** Correlation heatmap between immune cells; **(C)** Differential analysis of immune cell infiltration proportions between CRSwNP and control samples; **(D)** Correlation heatmap between key genes and immune cells. *P < 0.05, **P < 0.01, ***P < 0.001.

Immune infiltration analysis identified 22 types of immune cells in control and CRSwNP samples, with resting memory CD4 T cells accounting for the highest proportion in all samples (**Figure 5A**). Further exploration revealed the strongest positive correlation between activated NK cells and resting mast cells (r=0.445, P < 0.001), and the strongest negative correlation between naive B cells and plasma cells (r=-0.571, P < 0.001) (**Figure 5B**). Additionally, neutrophils, monocytes, M2 macrophages, and activated dendritic cells were significantly upregulated in CRSwNP samples (P < 0.05); while immune cells such as resting memory CD4 T cells and Plasma cells were significantly downregulated in CRSwNP samples (P < 0.05) (**Figure 5C**). Furthermore, both IL4R and IMPA2 were positively correlated with activated dendritic cells, M2 macrophages, and regulatory T cells (Tregs); and negatively correlated with plasma cells, resting memory CD4 T cells, and M1 macrophages. Additionally, PRR4 was positively correlated with resting memory CD4 T cells and activated mast cells; and negatively correlated with M2 macrophages, activated dendritic cells, and neutrophils (**Figure 5D**). The roles of these genes in immune cell infiltration may help reveal their potential mechanisms in CRSwNP.

### 3.8 Regulatory networks involving key genes

**Figure 6:**
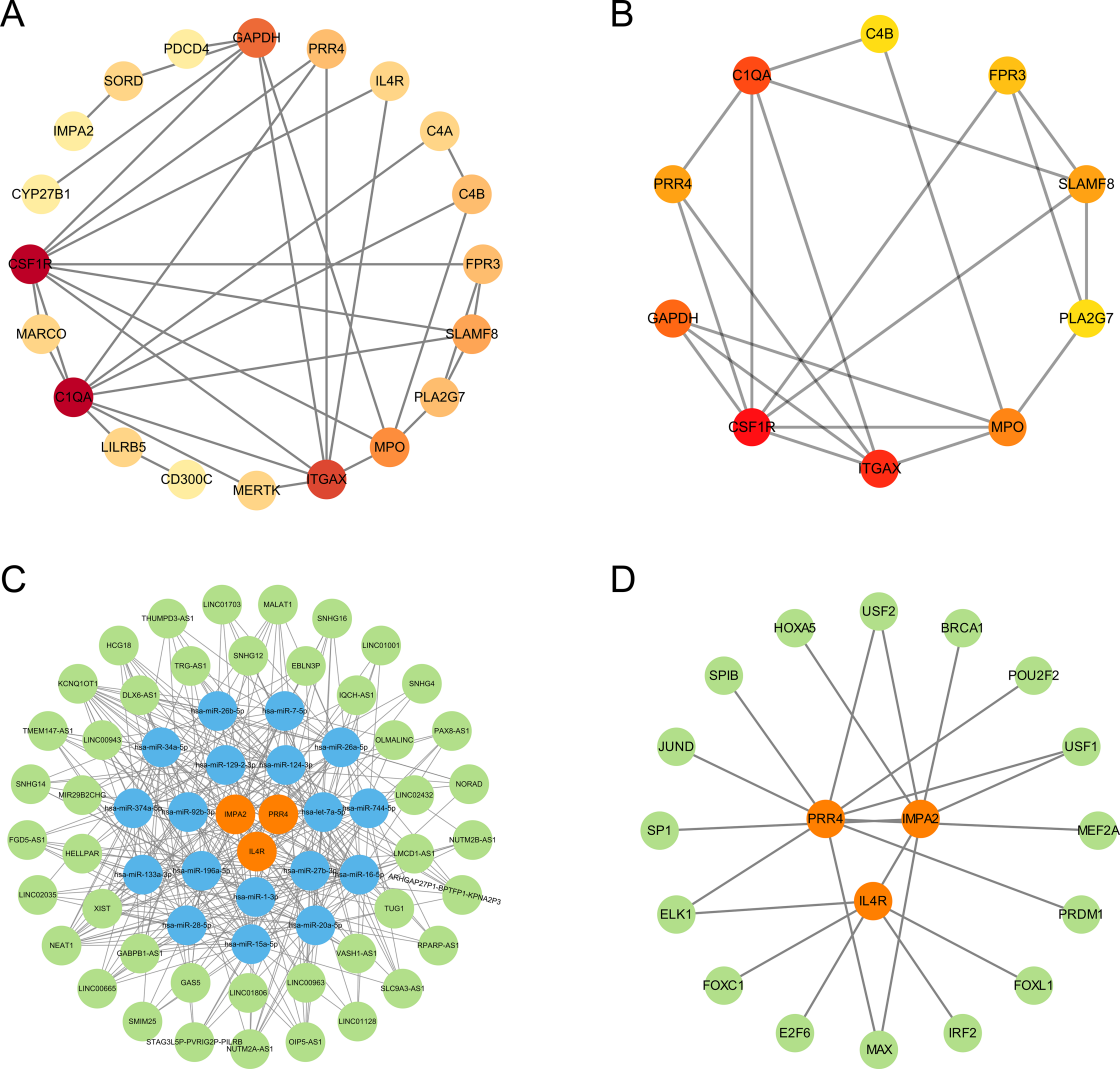
ceRNA network construction and transcription factor prediction. **(A)** PPI network analysis of candidate genes; **(B)** Top 10 genes screened by the cytoHubbA algorithm; **(C)** ceRNA network construction; **(D)** Transcription factor regulatory network.

PPI analysis of candidate genes is shown in **Figure 6A**. In the PPI network, there were 20 nodes and 34 edges, meaning 34 interaction relationships between 20 proteins. Among them, two proteins had direct interaction relationships with IL4R, only one protein had a direct interaction relationship with IMPA2, and three nodes had direct interaction relationships with PRR4, indicating that these key genes may directly or indirectly regulate these genes in the development of CRSwNP. Subsequently, the top 10 genes screened by the cytoHubbA algorithm, including CSF1R, ITGAX, and SLAMF8, had close interaction relationships with other proteins (**Figure 6B**).

In addition, the ceRNA network contained 64 nodes (including 3 key mRNAs, 18 miRNAs, and 43 lncRNAs) and 310 edges (interaction relationships). Among them, KCNQ1OT1 was the lncRNA with the highest topological parameter (Degree value) in the ceRNA network; hsa-miR-16-5p and hsa-miR-15a-5p were the miRNAs with the highest Degree values in the ceRNA network (**Figure 6C**).

Next, we performed TF prediction for the 3 key genes. The results showed 16 potential TF binding sites were significantly enriched in the promoter regions of key genes, including MAX, ELK1, USF2, and USF1 (**Figure 6D**).

### 3.9 Drug prediction and molecular docking

Combining the prediction results from the CMAP database and DSigDB database, a total of 26 candidate small molecule drugs targeting key genes were screened: ajmaline, alprostadil, ampyrone, anisomycin, budesonide, chlorhexidine, chlortetracycline, chlorzoxazone, clopamide, danazol, diphenylpyraline, dirithromycin, emetine, luteolin, mebendazole, methylprednisolone, metronidazole, progesterone, puromycin, quercetin, quinpirole, raloxifene, scriptaid, simvastatin, tamoxifen, and testosterone.

**Figure 7:**
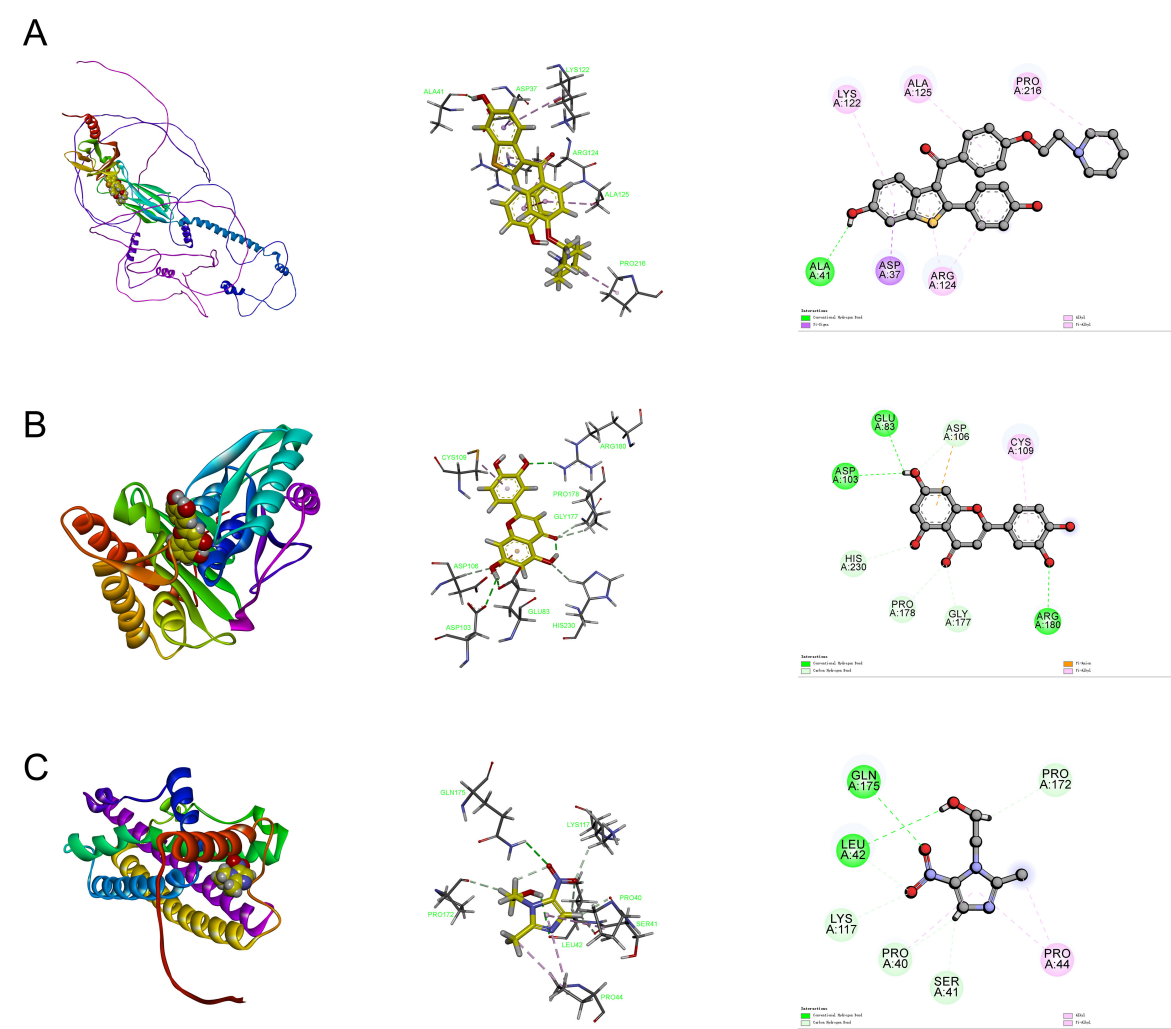
Molecular docking analysis of small molecule drugs and key targets. **(A–C)** Visualization of the best “ligand-receptor” complexes for IL4R **(A)**, IMPA2 **(B)**, and PRR4 **(C)**, showing binding interactions between small molecule drugs and target proteins.

Additionally, all small molecule drugs had good interaction relationships with key targets, with binding energies all less than -4kcal/mol (**Supplementary Table 3**). For key targets, the ligand raloxifene had the strongest targeting effect on IL4R (binding energy=-7.209 kcal/mol), forming hydrogen bonds and hydrophobic non-covalent bonds through the active pocket regions ALA41, ASP37, ARG124, LYS122, ALA125, and PRO216 of IL4R (**Figure 7A**). The ligand luteolin had the strongest targeting effect on IMPA2 (binding energy=-8.73 kcal/mol), forming hydrogen bonds, carbon-hydrogen bonds, and hydrophobic non-covalent bonds through the active pocket regions ASP103, GLU83, ASP106, GYS109, HIS230, PRO178, GLY177, and ARG180 of IMPA2 (**Figure 7B**). The ligand metronidazole had the strongest targeting effect on PRR4 (binding energy=-5.314 kcal/mol), forming hydrogen bonds, carbon-hydrogen bonds, and hydrophobic non-covalent bonds through the active pocket regions LEU42, GLN175, PRO172, LYS117, PRO40, SER41, and PRO44 of PRR4 (**Figure 7C**).

### 3.10 Expression validation and ROC curve analysis of key genes

**Figure 8:**
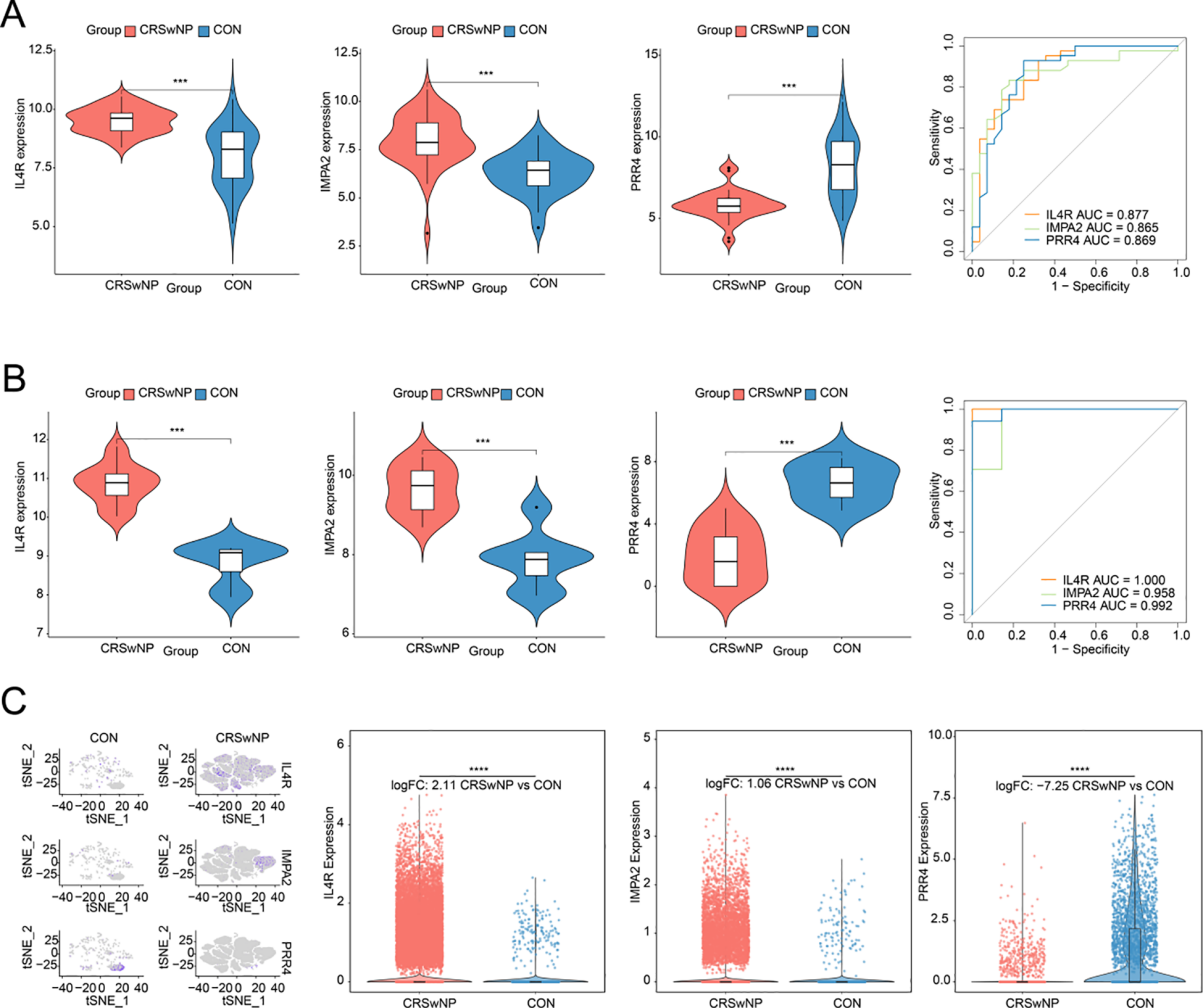
Expression validation and ROC analysis of key genes**. (A, B)** Expression analysis and ROC analysis of key genes IL4R, IMPA2, and PRR4 in the training set **(A)** and validation set **(B)**; **(C)** Expression characteristics and differences of key genes IL4R, IMPA2, and PRR4 in different groups. ***P < 0.001,****P < 0.0001.

In both the training and validation sets, compared to control group, IL4R and IMPA2 were significantly upregulated in the CRSwNP group (P < 0.05), while PRR4 was significantly downregulated, and the AUC values of the three key genes in both the training and validation sets were greater than 0.8 (**Figures 8A, B**), indicating that the key genes have good diagnostic capabilities. Moreover, at the single-cell level, the expression trends of the three key genes in the CRSwNP group and control group were consistent with the results in the bulk transcriptome (**Figure 8C**).

### 3.11 MR validation and co-localization analysis

IL4R (OR = 1.00127, 95%CI: 1.00003-1.00251, P = 0.04335) and IMPA2 (OR = 1.0005, 95%CI: 1.00001-1.00103, P = 0.04508) are risk factors for CRSwNP, while PRR4 (OR = 0.99951, 95%CI: 0.99905-0.99998, P = 0.04280) is a protective factor for CRSwNP (**Supplementary Figure 3A**). The scatter plot displays various results including MR Egger, weighted median, inverse variance weighted, simple mode, and weighted mode, with intercepts all near 0, and slopes positive for IL4R and IMPA2 but negative for PRR4, further confirming the robustness and reliability of the results (**Supplementary Figure 3B**). The funnel plot shows that the effect estimates (βIV) of each SNP are generally consistent with the IVW estimation line (**Supplementary Figure 3C**). Leave-one-out analysis found that the results do not depend on the contribution of any single SNP, further supporting the reliability of the causal relationship between IL4R, IMPA2, and PRR4 gene expression and the risk of CRSwNP (**Supplementary Figure 3D**).

Finally, co-localization analysis results showed that for the IL4R gene, the posterior probability of hypothesis H2 (only chronic rhinosinusitis has genetic association) was 0.516, the posterior probability of hypothesis H3 was 0.407, and the posterior probability of hypothesis H4 was 0.078. The analysis results for the IMPA2 gene showed that the posterior probability of hypothesis H2 was 0.659, the posterior probability of hypothesis H3 was 0.303, and the posterior probability of hypothesis H4 was 0.037. The analysis results for the PRR4 gene indicated that the posterior probability of hypothesis H2 was 0.649, the posterior probability of hypothesis H3 was 0.302, and the posterior probability of hypothesis H4 was 0.049 (**Supplementary Figure 3E**; **Supplementary Table 4**). These results suggest that the PPH4 values of all three genes (IL4R: 0.078; IMPA2: 0.037; PRR4: 0.049) are well below the standard threshold (PPH4 > 0.8) required to support shared causal variants, with H2 values predominating (0.516–0.659), indicating that the genetic association at these loci is likely trait-specific. Therefore, the current colocalization evidence is insufficient to support strong colocalization conclusions, and these results should be interpreted cautiously in conjunction with other multi-omics evidence.

### 3.12 Experimental validation

#### 3.12.1 Selecting optimal LPS concentration

To determine the optimal LPS concentration for establishing the *in vitro* CRSwNP model, HNEpC were treated with various concentrations of LPS (0.125-4 μg/mL) for 6 hours. As shown in **Supplementary Figure 4**, we selected 1 μg/mL LPS as the optimal concentration, which effectively induced inflammatory responses while preserving sufficient cell viability for subsequent functional assays.

#### 3.12.2 Effects of LPS on cell apoptosis, inflammatory cytokine expression, and tight junction proteins

**Figure 9:**
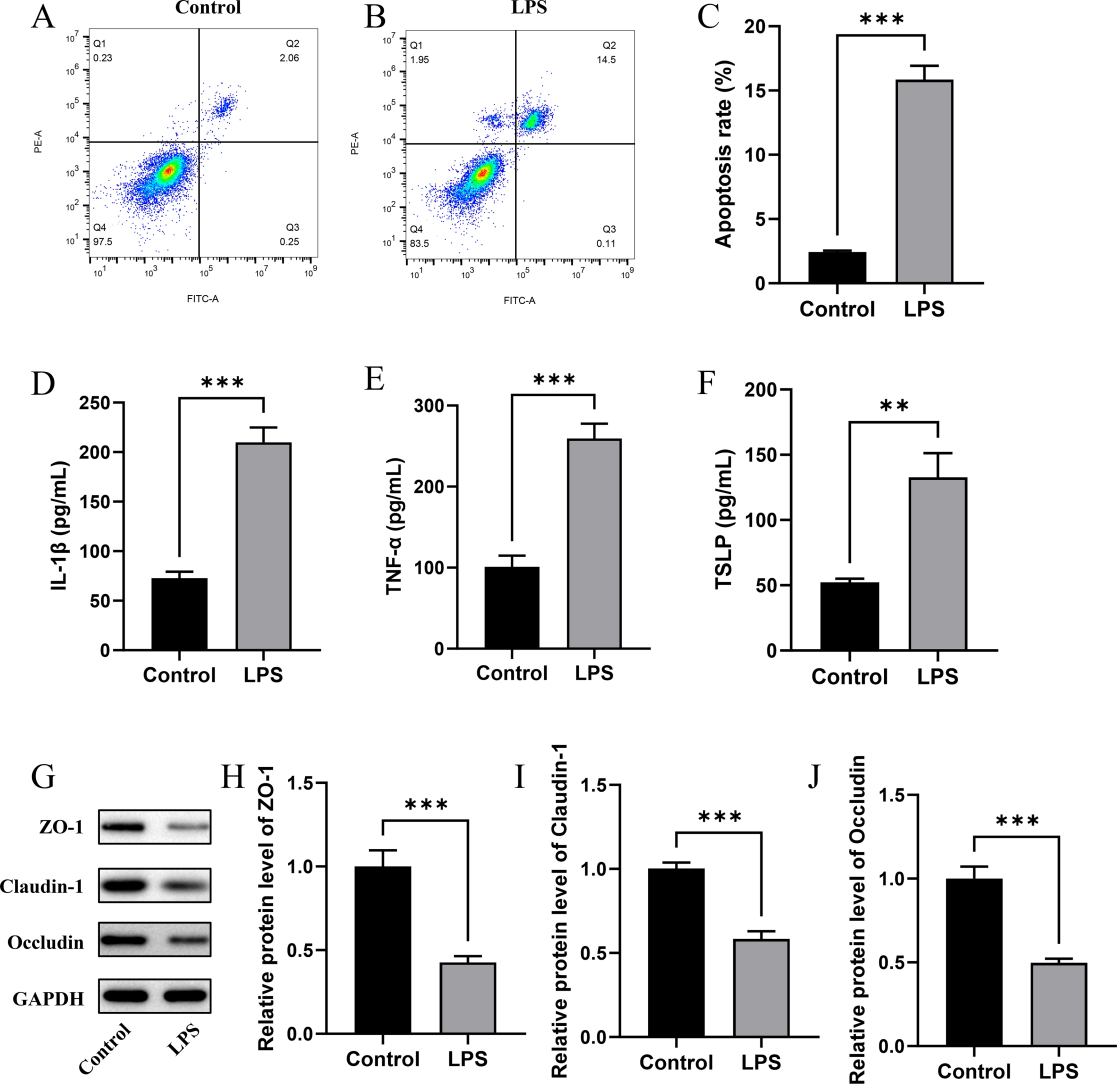
Effects of LPS on cell apoptosis, inflammatory cytokine expression, and tight junction proteins. **(A, B)** Flow cytometry plots showing apoptosis in control **(A)** and LPS **(B)** groups. **(C)** Quantification of total apoptosis. **(D–F)** qRT-PCR analysis of IL-1β **(D)**, TNF-α **(E)**, and TSLP **(F)** mRNA expression. **(G–J)** Western blot **(G)** and densitometric analyses of tight junction proteins: representative bands for ZO-1 **(H)**, Claudin-1 **(I)**, and Occludin **(J)** and their relative expression levels. ***P < 0.0001, **P < 0.01.

To investigate the effects of LPS on cell apoptosis, inflammatory response, and epithelial barrier integrity, flow cytometry, qRT-PCR, and Western blot analyses were performed. Compared with the control group, LPS treatment significantly induced cell apoptosis and triggered inflammatory responses, accompanied by disruption of epithelial tight junctions. Flow cytometry results showed a higher total apoptosis rate (P < 0.001) (**Figures 9A–C**). qRT-PCR analysis demonstrated that LPS markedly upregulated the mRNA expression of IL-1β, TNF-α, and TSLP (P < 0.01) (**Figures 9D–F**). Western blot results revealed that the expression levels of tight junction proteins ZO-1, Claudin-1, and Occludin were decreased after LPS exposure, and densitometric analysis confirmed significant reductions compared with the control group (P < 0.001) (**Figures 9G–J**). These findings indicate that LPS disrupts cellular integrity by inducing apoptosis and inflammation while downregulating tight junction proteins.

#### 3.12.3 Validation of IL4R and IMPA2 knockdown and PRR4 overexpression

To confirm the efficiency of gene knockdown and overexpression, the mRNA and protein levels of IL4R, IMPA2, and PRR4 were examined by qRT-PCR and Western blot. Compared with the negative control (NC) group, both mRNA and protein expression of IL4R (**Supplementary Figures 5A–C**) and IMPA2 (**Supplementary Figures 5D–F**) were significantly reduced in the si-IL4R and si-IMPA2 groups, respectively, while PRR4 expression (**Supplementary Figures 5G–I**) was markedly increased in the oe-PRR4 group (P < 0.01). These results indicate that the knockdown of IL4R and IMPA2 and the overexpression of PRR4 were successfully achieved.

#### 3.12.4 Effects of IL4R, IMPA2, and PRR4 modulation on LPS-induced injury in HNEpC cells

**Figure 10:**
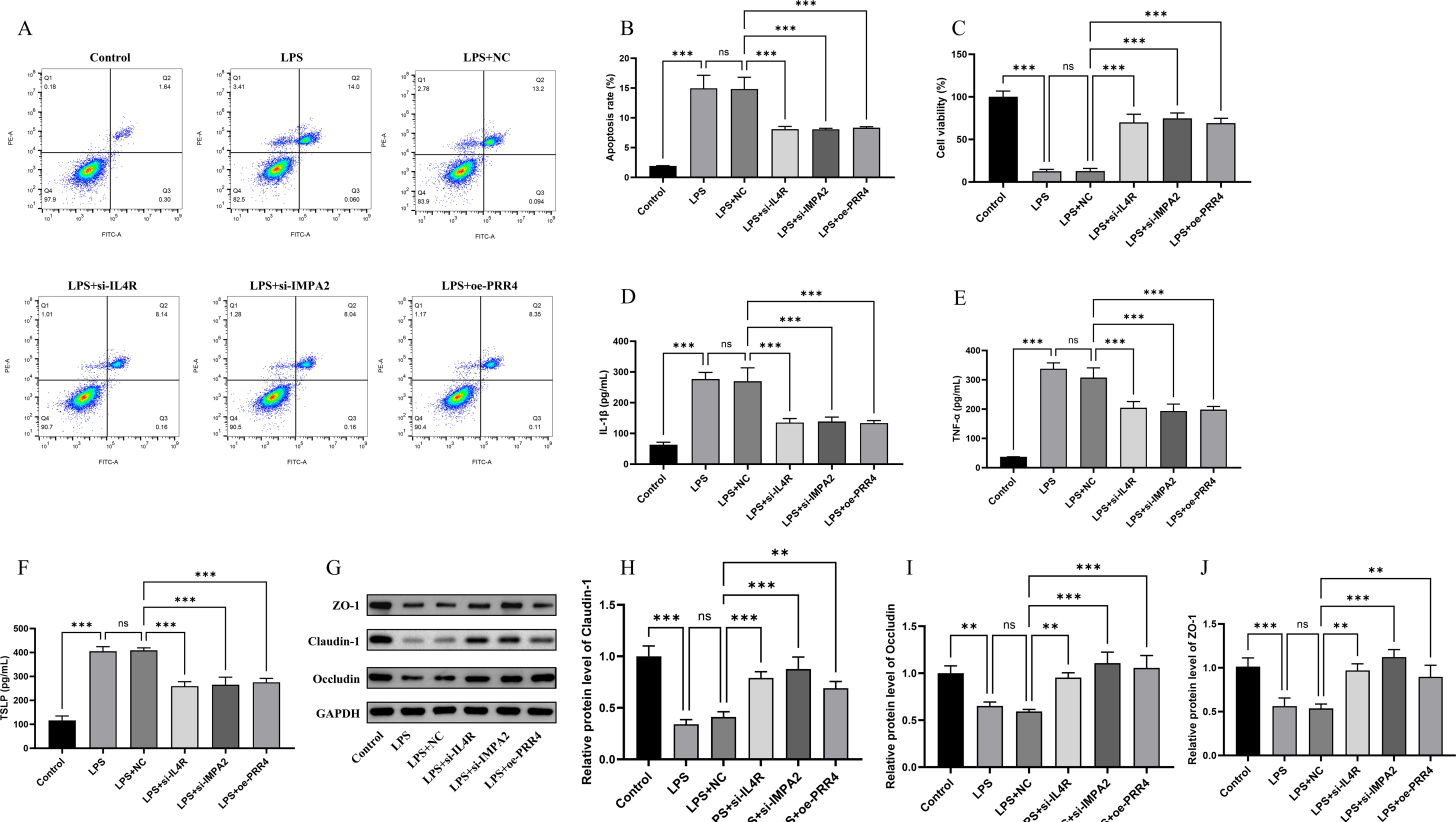
Effects of IL4R, IMPA2, and PRR4 modulation on LPS-induced injury in HNEpC cells. **(A, B)** Flow cytometry analysis of cell apoptosis. Representative scatter plots of different treatment groups **(A)** and quantification of apoptosis rates **(B)**. **(C)** Cell viability detected by CCK-8 assay. **(D–F)** ELISA quantification of inflammatory cytokines IL-1β **(D)**, TNF-α **(E)**, and TSLP **(F)** levels in cell culture supernatants. **(G–J)** Western blot analysis of tight junction proteins. Representative blots **(G)** and quantification of ZO-1 **(H)**, Claudin-1 **(I)**, and Occludin **(J)** protein expression levels normalized to GAPDH. ***P < 0.0001, **P < 0.01; ns, not significant.

To further investigate the roles of IL4R, IMPA2, and PRR4 in LPS-induced HNEpC injury, we performed gene knockdown and overexpression experiments. Flow cytometry analysis (**Figures 10A, B**) revealed that LPS treatment significantly increased the apoptosis rate compared to the untreated control group (P < 0.001). Notably, knockdown of IL4R or IMPA2, as well as overexpression of PRR4, all significantly reduced the apoptosis rate under LPS stimulation (P < 0.001). CCK8 assay results (**Figure 10C**) showed that LPS treatment significantly decreased cell viability to approximately 10% (P < 0.001). Knockdown of IL4R or IMPA2, as well as overexpression of PRR4, all significantly restored cell viability to approximately 70% (P < 0.001). ELISA analysis of inflammatory cytokines (**Figures 10D, F**) demonstrated that IL-1β, TNF-α, and TSLP secretion levels were significantly elevated following LPS stimulation (P < 0.001). Knockdown of IL4R or IMPA2, as well as overexpression of PRR4, all significantly suppressed the release of these three inflammatory cytokines (P < 0.001). Western blot analysis of tight junction proteins (**Figures 10G–J**) showed that LPS treatment resulted in significant downregulation of ZO-1, Claudin-1, and Occludin protein expression levels (P < 0.01). Knockdown of IL4R or IMPA2, as well as overexpression of PRR4, all significantly upregulated the expression of these tight junction proteins (P < 0.01), restoring epithelial barrier function. Collectively, these results indicate that knockdown of IL4R or IMPA2, as well as overexpression of PRR4, can effectively protect HNEpC cells from LPS-induced injury, attenuate inflammatory responses, and maintain epithelial barrier integrity.

## 4 Discussion

This study systematically identified the key molecular events and dysfunctions in CRSwNP by integrating single-cell sequencing, transcriptomic data, GWAS, and eQTL analysis, and clarified the important roles of three key genes, IL4R, IMPA2, and PRR4, in the development of CRSwNP. MR analysis preliminarily suggested that IL4R and IMPA2 may be potential risk factors for CRSwNP, while PRR4 may serve as a potential protective factor, though the effect sizes were modest (OR values close to 1) and P values were borderline significant (0.043–0.045), indicating limited evidence strength. At the same time, a regulatory network centered on these three key genes was constructed, and potential therapeutic drugs such as raloxifene, luteolin, and metronidazole were predicted, providing new ideas for the precise diagnosis and treatment of CRSwNP.

Through single-cell sequencing analysis, we found that fibroblasts were significantly increased in CRSwNP tissues and maintained extensive communication with various immune cells. This finding is consistent with previous studies, which found that stromal fibroblasts in CRSwNP exhibit a unique activated phenotype, involved in tissue remodeling and inflammation regulation (21). Moreover, another study further confirmed that fibroblasts secrete chemokines such as CCL11 and CCL24 to recruit eosinophils and other inflammatory cells, promoting type 2 inflammation (22). The cell communication network analysis in this study showed that fibroblasts interact closely with B cells, dendritic cells, and plasma cells, supporting the view that fibroblasts regulate the immune microenvironment through the secretion of cytokines and chemokines.

Interleukin-4 receptor (IL4R) is a key upregulated gene identified in this study, encoding the common receptor α chain for IL-4 and IL-13, playing a core role in the type 2 inflammatory response (23). Our Mendelian randomization analysis preliminarily suggested that upregulation of IL4R expression is a risk factor for CRSwNP (OR = 1.00127, P = 0.04335); however, the effect size was modest and the P value borderline significant, so this finding should be interpreted as indicative rather than conclusive. A study found that IL4R gene polymorphism is significantly associated with asthma susceptibility (24), and this study further confirmed the risk association between this gene and CRSwNP. Furthermore, the IL-4/IL-13 signaling pathway mediated by IL4R plays a key role in the pathophysiology of CRSwNP. Biological agents targeting IL4R, such as dupilumab, significantly improve clinical symptoms in CRSwNP patients, reducing nasal polyp size and recurrence rates (25).

Inositol monophosphatase 2 (IMPA2) plays an important role in inositol metabolism, which is closely related to cell signaling, inflammation response, and cell growth (26). Our Mendelian randomization analysis preliminarily suggested that upregulation of IMPA2 expression is a risk factor for CRSwNP (OR = 1.0005, P = 0.04508); given the near-unity OR and borderline P value, this causal inference requires verification in future studies using CRSwNP-specific GWAS data. Although the role of IMPA2 in CRSwNP has not been widely studied, existing evidence suggests that inositol metabolism disorders are associated with various inflammatory diseases. A study found that inositol metabolites are significantly altered in the fibroblasts of patients with idiopathic pulmonary fibrosis, and inositol treatment can reduce the invasiveness of IPF lung fibroblasts (27). Proline-rich protein 4 (PRR4) is mainly expressed in lacrimal gland tissue and mucosal epithelium, exhibiting antimicrobial and anti-inflammatory functions (28). MR analysis confirmed that downregulation of PRR4 expression is a risk factor for CRSwNP (OR = 0.99951, P = 0.04280), with the caveat that the very small effect size and borderline significance indicate limited evidence strength at present. Studies have shown that PRR4 is abundantly expressed in healthy nasal mucosal epithelium, inhibiting bacterial biofilm formation and the release of inflammatory mediators, with its expression significantly downregulated in the CRSwNP group (29), consistent with the findings of this study. In conclusion, the key genes identified in this study play significant roles in CRSwNP, and these genes may serve as new targets for genetic risk assessment and targeted therapy for CRSwNP. Future research should focus on the functional validation and clinical translation of these genes, including genotype-based disease risk prediction, drug response evaluation, and the development of personalized treatment strategies.

The relationship between fibroblast biology and the three key genes identified in this study merits further discussion. IL-4 and IL-13 are two key cytokines playing a pivotal role in CRSwNP, both acting through the alpha subunit of the same receptor (IL4R) (30). IL-4 gives priority to activating Th2 cells, macrophages, and fibroblasts, while its downstream effects are associated with IgE isotype switching, Th2 cell differentiation, and tissue remodeling (31). Specifically, IL-4 induced changes in mRNA and protein expression of fibrotic mediators (including TGF-β1 and TGF-β2) and inflammatory chemokines (including IL-6 and CCL11) in fibroblasts isolated from nasal polyp tissue, all of which expressed the IL-4 receptor (32). Furthermore, Steinke et al. reported that nasal polyp-derived fibroblasts (NPDF) express IL-4R, and that receptor activation upregulates TGF-β1, IL-6, CCL11 (eotaxin-1), and CCL13 (MCP-4) (33). CRSwNP is associated with inflammation and tissue remodeling including myofibroblast differentiation and extracellular matrix (ECM) deposition, to which both TGF-β1 and IL-4 have been shown to contribute as key profibrotic mediators (34). The IL4R upregulation identified in the present study may therefore reflect not only immune and epithelial dysregulation but also fibroblast-driven stromal remodeling as an additional pathological axis.

Regarding IMPA2, inositol monophosphatase activity has been shown to regulate fibroblast invasiveness in IPF: IMPA2 mRNA was markedly reduced in IPF lung fibroblasts, and inositol supplementation suppressed ASS1-mediated profibrotic signaling, reduced cell invasiveness, and attenuated bleomycin-induced fibrosis *in vivo (*27). By analogy, IMPA2 dysregulation in CRSwNP may similarly perturb inositol-dependent signaling, contributing to aberrant fibroblast activation and stromal remodeling. With respect to PRR4, its downregulation in CRSwNP may compromise mucosal innate defense, creating a permissive inflammatory microenvironment that indirectly sustains fibroblast activation and tissue remodeling chronicity. These proposed associations remain inferential, derived from transcriptomic data and cross-disease extrapolation rather than direct experimental validation. Future studies utilizing single-cell RNA sequencing, functional perturbation assays, or co-culture models in CRSwNP-derived fibroblast populations will be necessary to establish the functional contributions of IL4R, IMPA2, and PRR4 to stromal remodeling in CRSwNP.

The immune infiltration analysis in this study showed a significant increase in monocytes, M2 macrophages, and neutrophils in CRSwNP tissues, while resting memory CD4 T cells were significantly decreased. This is consistent with previous reports and emphasizes the importance of immune cells in the pathological process of CRSwNP. The increased neutrophils observed in our study were consistent with previous research, which reported that neutrophils enhance inflammation and tissue damage by releasing neutrophil extracellular traps (NETs) (35). Notably, the positive correlation between IL4R expression and M2 macrophage infiltration reflects a direct signaling relationship: IL-4 binding to IL4R engages JAK1 and TYK2 kinases to phosphorylate STAT6, which serves as the canonical transcriptional driver of M2 macrophage polarization, upregulating anti-inflammatory mediators including IL-10, CCL18, and CD206 (36). Once polarized, M2 macrophages amplify type 2 inflammation by secreting CCL18 and IL-10 (3), while further upregulating IL4R on neighboring epithelial and stromal cells, potentially establishing a self-reinforcing inflammatory loop that perpetuates polyp growth. The concurrent positive correlation of IL4R with regulatory T cells (Tregs) likely reflects the tolerogenic immune adaptation inherent to chronic type 2 inflammation, wherein sustained IL-4/IL-13 signaling fosters an immunosuppressive microenvironment by promoting Treg expansion through STAT6-dependent mechanisms (37). Correspondingly, the observed depletion of resting memory CD4 T cells may reflect progressive Th2 polarization bias driven by chronic IL4R activation, redirecting CD4 T cell differentiation away from memory maintenance toward Th2 effector fates (38). Regarding IMPA2, its upregulation and positive correlation with M2 macrophage infiltration may reflect its modulatory role in the inositol phosphate signaling cascade. IMPA2 overexpression in CRSwNP may therefore prime immune cells toward M2-skewed responses by augmenting PI3K-dependent second messenger flux. The negative correlation between PRR4 and neutrophil infiltration is consistent with PRR4’s established function as a mucosal antimicrobial and anti-inflammatory factor. PRR4 downregulation in CRSwNP may permit increased microbial colonization and biofilm persistence, generating sustained pathogen-associated molecular patterns (PAMPs) that activate TLR4/NF-κB signaling in epithelial cells and thereby drive the production of neutrophil-recruiting chemokines, particularly CXCL8. These findings collectively reveal that key genes participate in the pathogenesis of CRSwNP by regulating the immune microenvironment.

Through integrative analysis, we constructed a ceRNA regulatory network centered on IL4R, IMPA2, and PRR4 and found that the lncRNA KCNQ1OT1, as a key regulatory factor, may regulate IL4R expression by competitively binding miR-16-5p and miR-15a-5p. KCNQ1OT1 is upregulated in the airway epithelium of asthma patients and regulates downstream inflammatory-related gene expression by sponge adsorption of miRNAs (39). Our transcription factor prediction analysis identified key transcription factors such as GATA2, MAX, ELK1, USF1, and USF2. Among them, GATA2 has been shown to regulate the expression of various inflammatory factors and chemokines (40). These findings provide important clues for further understanding the transcriptional regulation mechanisms of key genes.

Based on the expression characteristics of key genes, this study predicted 26 potential therapeutic drugs, among which raloxifene, luteolin, and metronidazole showed good binding affinity with IL4R, IMPA2, and PRR4, respectively. Raloxifene, a selective estrogen receptor modulator, has been found to have immunoregulatory effects in recent years (41). Studies have shown that raloxifene can inhibit STAT6 phosphorylation, reduce the activity of the IL-4/IL-13 signaling pathway, and thereby decrease the type 2 inflammatory response (42). Given the key role of IL4R in CRSwNP, raloxifene may be a potential therapeutic option. Existing studies suggest that luteolin has broad anti-inflammatory and antioxidant effects, inhibiting NF-κB and MAPK signaling pathways, reducing the production of inflammatory factors, and improving airway inflammation (43). Although traditionally recognized as an antiprotozoal and antibacterial agent, emerging evidence indicates that metronidazole possesses anti-inflammatory properties through suppression of NF-κB-mediated inflammatory mediator production (44). Molecular docking confirmed binding to the PRR4 active pocket via residues LEU42, GLN175, PRO172, LYS117, PRO40, SER41, and PRO44, suggesting it may stabilize PRR4 conformation and potentially enhance its mucosal anti-inflammatory function. We acknowledge that the direct mechanistic basis for metronidazole’s interaction with PRR4 requires experimental validation, and these predictions are presented as hypothesis-generating.

Our study found that LPS stimulation significantly induced HNEpC cell apoptosis, promoted the release of inflammatory cytokines (IL-1β, TNF-α, TSLP), and disrupted the expression of tight junction proteins (ZO-1, Claudin-1, Occludin). Epithelial barrier dysfunction is considered one of the core mechanisms in the pathogenesis of CRSwNP (45, 46). Tight junction proteins are key structural components maintaining epithelial barrier integrity, and their downregulation leads to impaired barrier function, increasing the permeability to allergens and pathogens, thereby exacerbating inflammatory responses (47, 48). Studies have shown that type 2 inflammatory cytokines such as IL-13 can disrupt tight junctions by downregulating the expression of ZO-1 and Occludin (49, 50), which is consistent with our observation of LPS-induced reduction in tight junction protein expression. Notably, TSLP, as an epithelial-derived cytokine highly expressed in CRSwNP, is a key regulator initiating type 2 inflammatory responses (51, 52). TSLP can activate dendritic cells to induce Th2 cell differentiation, promote eosinophil recruitment, and continuously amplify inflammatory responses through positive feedback mechanisms (53, 54). Our results showed that LPS significantly upregulated TSLP mRNA expression, which may represent an important link between inflammatory stimuli and type 2 immune responses, suggesting that epithelial cells act as a bridge in the transition from infection-related inflammation to type 2 inflammation.

More importantly, our functional validation experiments confirmed that knockdown of IL4R and IMPA2, as well as overexpression of PRR4, all significantly attenuated LPS-induced cell injury. Specifically, these gene modifications significantly reduced apoptosis rates, improved cell viability, suppressed inflammatory cytokine release, and restored tight junction protein expression. These results directly confirm at the cellular functional level the roles of IL4R and IMPA2 in promoting inflammatory responses in CRSwNP, as well as the protective role of PRR4. The IL-4/IL-13 signaling pathway mediated by IL4R is central to type 2 inflammation, and studies have shown that overactivation of this pathway can induce epithelial-to-mesenchymal transition (EMT) in epithelial cells, promoting tissue remodeling and polyp formation (55). Our results showing that IL4R knockdown can attenuate inflammatory responses and restore barrier function provide mechanistic support for the effectiveness of IL4R-targeting biologics (such as dupilumab) in CRSwNP treatment (25). Although the role of IMPA2 in CRSwNP has not been extensively studied, our experimental results indicate that its knockdown also has anti-inflammatory and barrier-protective effects, suggesting that IMPA2 may participate in inflammation regulation by affecting inositol metabolism-related signaling pathways (27). PRR4, as a protein with antimicrobial and anti-inflammatory functions, showed significant improvement in LPS-induced cell injury upon overexpression, consistent with its role in maintaining mucosal homeostasis and inhibiting inflammatory mediator release (29).

The choice of LPS as the inflammatory stimulus in our *in vitro* model requires clarification. CRSwNP is a heterogeneous disease comprising distinct endotypes with different immunologic profiles. Although eosinophilic/Th2-dominant phenotypes predominate in Western populations, non-T2 inflammation CRSwNP characterized by prominent neutrophil infiltration accounts for about half of cases in East Asian populations, including Chinese patients (56, 57). Furthermore, a study has shown that the LPS-induced neutrophil nasal polyp model closely matches the primary nasal polyp characteristics identified in the Asian population, providing a highly relevant model for investigating the pathogenesis of CRSwNP in this population (58). Notably, our own immune infiltration analysis revealed that neutrophils, monocytes, and M2 macrophages were significantly elevated in CRSwNP samples, consistent with a non-T2/neutrophilic inflammatory profile that corroborates our choice of LPS as a disease-relevant stimulus. Therefore, in this study, LPS was used to stimulate HNEpC cells, aiming to simulate the mechanism of nasal polyps that are similar to those of the Asian population and are mainly composed of neutrophils.

In addition, one of the core functional experiments of this study was the verification of IL4R knockdown in the LPS + si-IL4R group. IL4R encodes the common receptor α subunit (IL-4Rα) for both IL-4 and IL-13, serving as the essential shared binding chain and the sole receptor component required for initiating downstream signaling cascades of both cytokines (59). In contrast, LPS acts primarily through the TLR4 in nasal epithelial cells and does not involve direct receptor-level cross-talk with IL4R (60, 61). Knocking down IL4R in the LPS-stimulated context can therefore objectively reflect the independent functional contribution of IL4R to epithelial cell inflammatory stress responses, beyond its classical role in Th2 cytokine signaling. However, that direct validation of IL4R’s Th2-specific functions would be better achieved using IL-4/IL-13 stimulation models. Such comparative or combined endotype-specific experiments represent an important direction for future studies.

In summary, our *in vitro* functional validation experiments not only confirmed the key roles of IL4R, IMPA2, and PRR4 in the pathogenesis of CRSwNP but also revealed how these genes affect disease progression by regulating cell apoptosis, inflammatory responses, and epithelial barrier integrity. These findings provide important experimental evidence for understanding the molecular mechanisms of CRSwNP and offer a theoretical basis for developing new therapeutic strategies.

Several limitations of this study should be acknowledged. First, the GWAS dataset used in the MR analysis (ukb-a-541) represents a broad chronic rhinosinusitis phenotype rather than CRSwNP specifically, which introduces phenotypic heterogeneity and may limit the specificity of the causal inferences drawn. Combined with the modest effect sizes (OR values close to 1) and borderline P values (0.043–0.045), the strength of the current MR evidence is limited, and future studies utilizing CRSwNP-specific GWAS data are warranted. Second, the colocalization analysis revealed that PPH4 values for all three key genes (IL4R: 0.078; IMPA2: 0.037; PRR4: 0.049) were substantially below the conventional threshold of 0.8, indicating insufficient evidence for shared causal variants between gene expression and CRSwNP risk. These colocalization results should therefore be interpreted cautiously and only in conjunction with other multi-omics evidence. Third, the single-cell dataset (GSE276503) included only 2 control samples compared to 15 CRSwNP samples, representing a considerable imbalance that may affect the reliability of between-group comparisons at the single-cell level. Furthermore, the single-cell data were not directly incorporated into the gene screening pipeline, and the CIBERSORTx immune deconvolution analysis and single-cell compositional analysis were presented as independent modules without systematic cross-validation; future studies should employ larger, more balanced single-cell cohorts and consider constructing disease-specific reference matrices to enable deeper multi-omics integration. Fourth, the ceRNA regulatory network and drug predictions generated in this study are purely computational analyses and have not been experimentally validated; these findings should be regarded as hypothesis-generating rather than definitive conclusions. Fifth, the present study lacks *in vivo* validation, and the causal roles of IL4R, IMPA2, and PRR4 in CRSwNP pathogenesis remain to be confirmed in animal models or clinical cohorts. Finally, the LPS-induced *in vitro* model used in this study primarily activates the TLR4/NF-κB signaling pathway, which more closely mimics innate immune activation and epithelial barrier disruption than the Th2-dominant inflammatory microenvironment that characterizes eosinophilic CRSwNP in Western populations. Although LPS effectively recapitulates key features of epithelial injury including apoptosis, inflammatory cytokine release, and tight junction protein downregulation, it does not fully reproduce the complex type 2 immune milieu relevant to eosinophilic CRSwNP. Therefore, while this model is well-suited for investigating the neutrophilic endotype predominant in East Asian populations, the Th2-specific functions of IL4R warrant further investigation using IL-4/IL-13 stimulation or patient-derived primary cell models in future studies.

## Data Availability

The original contributions presented in the study are included in the article/**Supplementary Material**. Further inquiries can be directed to the corresponding author.
