# Supplementary material for: Multi-omics integration and in vitro validation identify IL4R, IMPA2, and PRR4 as key therapeutic targets in chronic rhinosinusitis with nasal polyps
Source: Front Immunol. 2026 Jun 15;17:1792878. doi: 10.3389/fimmu.2026.1792878 (PMC13310663; doi:10.3389/fimmu.2026.1792878)
Supplement: Supplementary file 3 [file DataSheet3.docx]

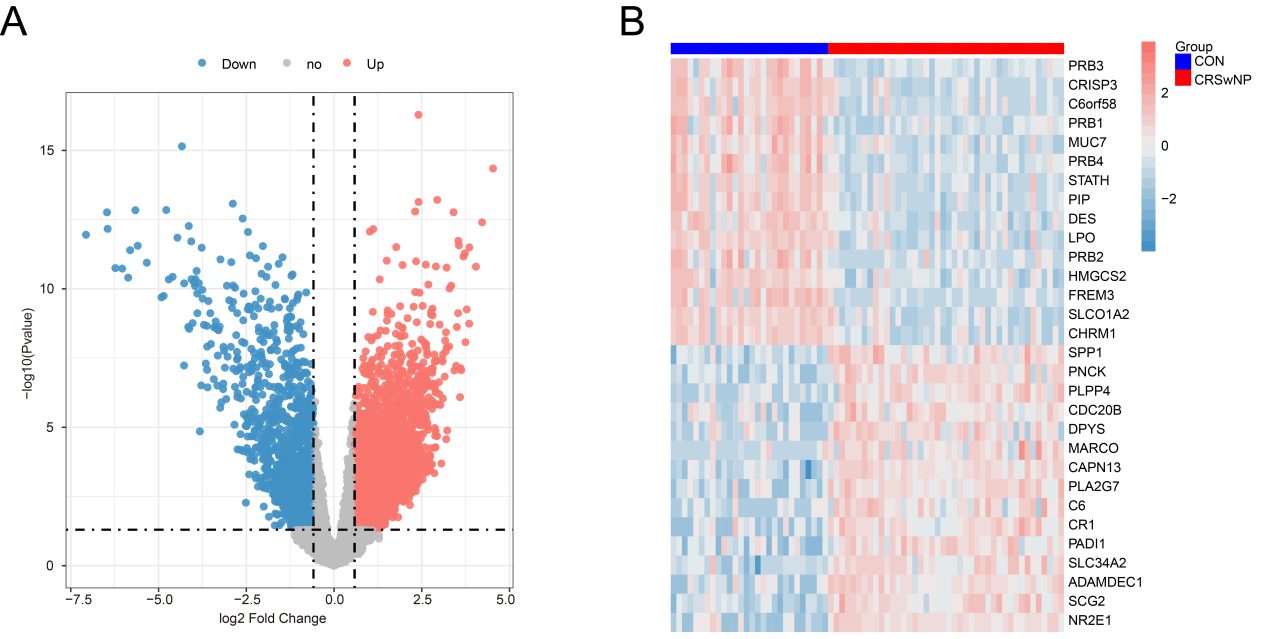
**Supplementary Figures 1 Transcriptome data expression differences between CRSwNP tissue and control tissue**

1. Volcano plot of differential expression analysis; (B) Heatmap of the top 15 upregulated and downregulated differential genes.


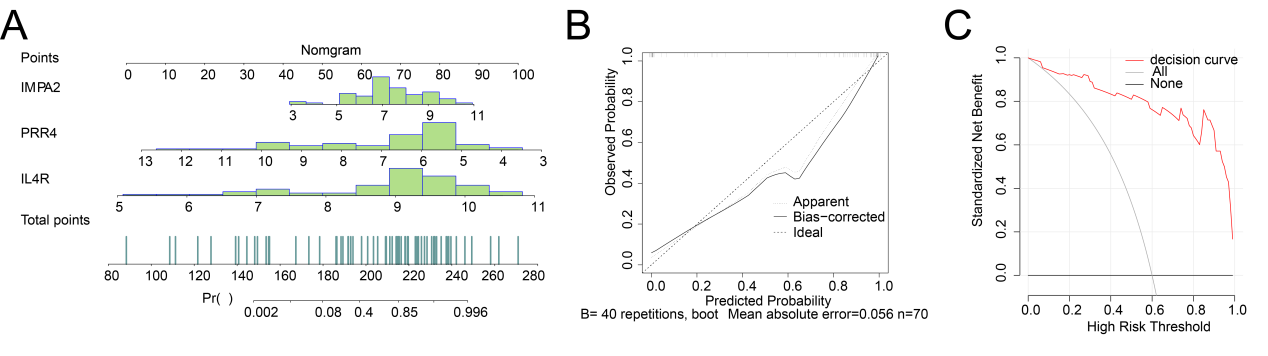
**Supplementary Figures 2 Nomogram and evaluation results**

1. Nomogram model constructed with key genes; (B) Calibration curve evaluating model accuracy; (C) Decision curve of the model.

**Table S2. The primer sequences**

| **Genes** | **Forward primer (5'->3')** | **Reverse primer (5'->3')** |
| --- | --- | --- |
| IL4R | GCGTCTCCGACTACATGAGC | ATACACGTGTGGGCTTCGG |
| IMPA2 | CGGGCAGGACAGATCATCA | TGTGAAGGAAACCTCTCTCGC |
| PRR4 | CTCAGAGCACAGATAATGATGTGAA | GTCTCTGGGGTCCCTGATCT |
| GAPDH | AGGCCGGATGTGTTCGC | CAAATCCGTTGACTCCGACC |

| **Table S3 Binding free energies between key genes and small molecule drugs** |
| --- |

| Compound | Target | Binding free energies (-kcal/mol) |
| --- | --- | --- |
| alprostadil | IL4R | 4.956 |
| budesonide | IL4R | 5.681 |
| diphenylpyraline | IL4R | 6.148 |
| mebendazole | IL4R | 6.159 |
| methylprednisolone | IL4R | 5.719 |
| progesterone | IL4R | 6.119 |
| quercetin | IL4R | 5.647 |
| quinpirole | IL4R | 4.309 |
| raloxifene | IL4R | 7.209 |
| scriptaid | IL4R | 5.889 |
| simvastatin | IL4R | 6.806 |
| tamoxifen | IL4R | 5.005 |
| testosterone | IL4R | 6.02 |
| ajmaline | IMPA2 | 6.84 |
| ampyrone | IMPA2 | 5.009 |
| anisomycin | IMPA2 | 5.775 |
| chlorhexidine | IMPA2 | 7.95 |
| chlortetracycline | IMPA2 | 6.156 |
| chlorzoxazone | IMPA2 | 5.744 |
| clopamide | IMPA2 | 6.262 |
| danazol | IMPA2 | 7.285 |
| dirithromycin | IMPA2 | 6.362 |
| emetine | IMPA2 | 6.834 |
| luteolin | IMPA2 | 8.73 |
| progesterone | IMPA2 | 6.88 |
| puromycin | IMPA2 | 5.686 |
| quercetin | IMPA2 | 7.251 |
| metronidazole | PRR4 | 5.314 |

**
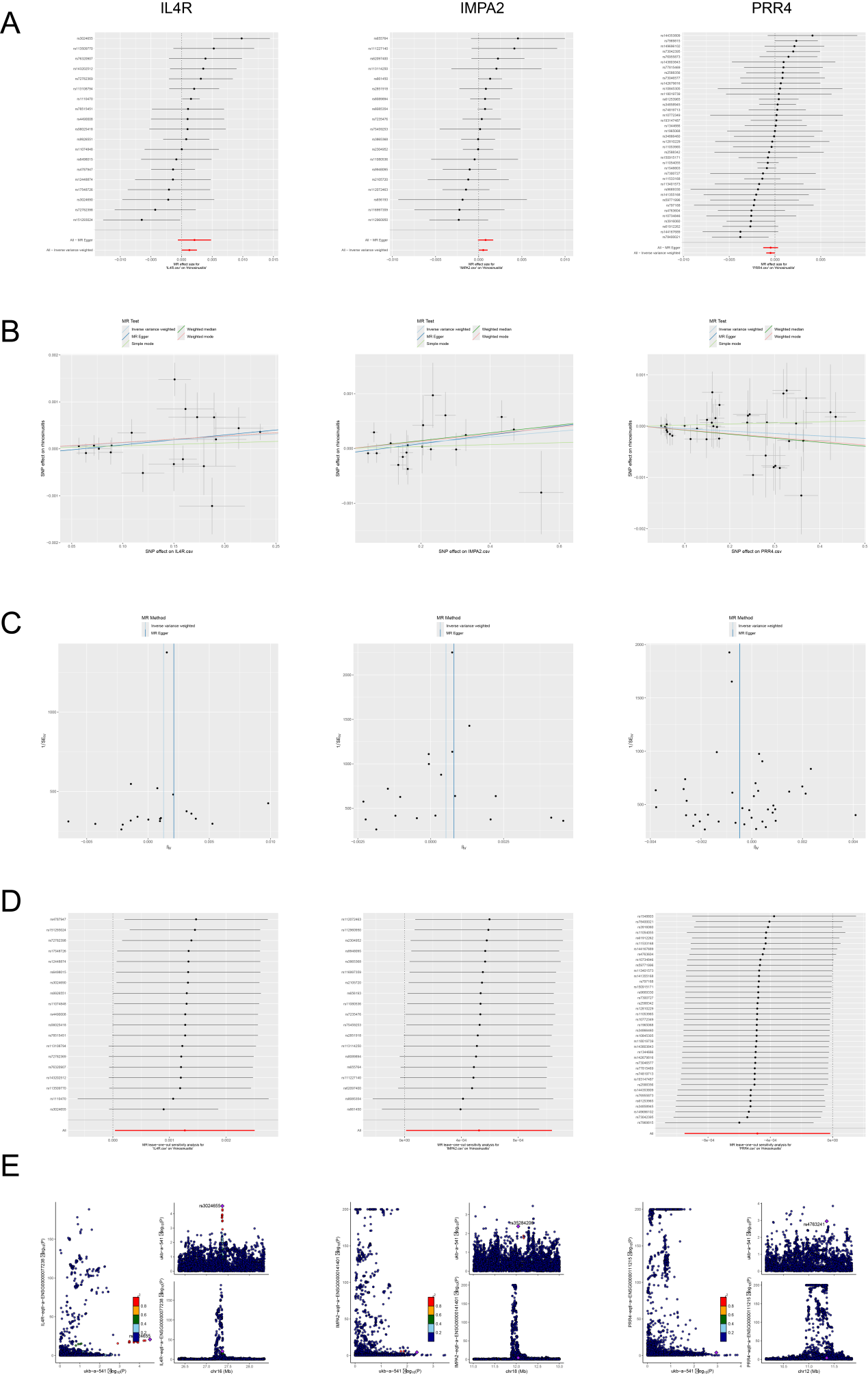
Supplementary Figure 3 Mendelian randomization analysis results of IL4R, IMPA2, and PRR4 genes with CRSwNP risk**

(A) Forest plot showing the effects of IL4R, IMPA2, and PRR4 gene eQTLs. (B) Scatter plot showing causal effect estimates of IL4R, IMPA2, and PRR4 gene expression levels on CRSwNP risk using various Mendelian randomization methods, used to evaluate consistency and robustness between methods. (C) Funnel plot for evaluating publication bias and heterogeneity of instrumental variable effects. (D) Leave-one-out sensitivity analysis plot for evaluating the influence of individual SNPs on overall causal effect results; stable effect values indicate that research results are not overly driven by single SNPs. (E) Co-localization analysis of IL4R, IMPA2, and PRR4 genes and CRSwNP.

**Table S4 Co-localization analysis**

|  | IL4R | IMPA2 | PRR4 |
| --- | --- | --- | --- |
| PP.H0.abf | 3.99E-182 | 2.55E-195 | 2.74E-196 |
| PP.H1.abf | 3.15E-182 | 1.18E-195 | 1.28E-196 |
| PP.H2.abf | 0.515796412 | 0.659449948 | 0.648737587 |
| PP.H3.abf | 0.406664537 | 0.303405624 | 0.301879426 |
| PP.H4.abf | 0.07753905 | 0.037144427 | 0.049382987 |

PP.H0.abf: Probability of no association with the gene or CRSwNP; PP.H1.abf: Probability of association only with the gene; PP.H2.abf: Probability of association only with CRSwNP; PP.H3.abf: Probability of genetic association with both the gene and CRSwNP, but caused by different genetic variants; PP.H4.abf: Probability of genetic association with both the gene and CRSwNP, and caused by the same genetic variant.

**Supplementary Figure 4 The optimal LPS concentration for establishing the *in vitro* CRSwNP model**

Cell viability of HNEpC cells treated with various concentrations of LPS (0.125, 0.25, 0.5, 1, 2, and 4 μg/mL) for 6 hours, as measured by CCK-8 assay. Data are presented as mean ± SD from three independent experiments. ***P < 0.001, ns = not significant.


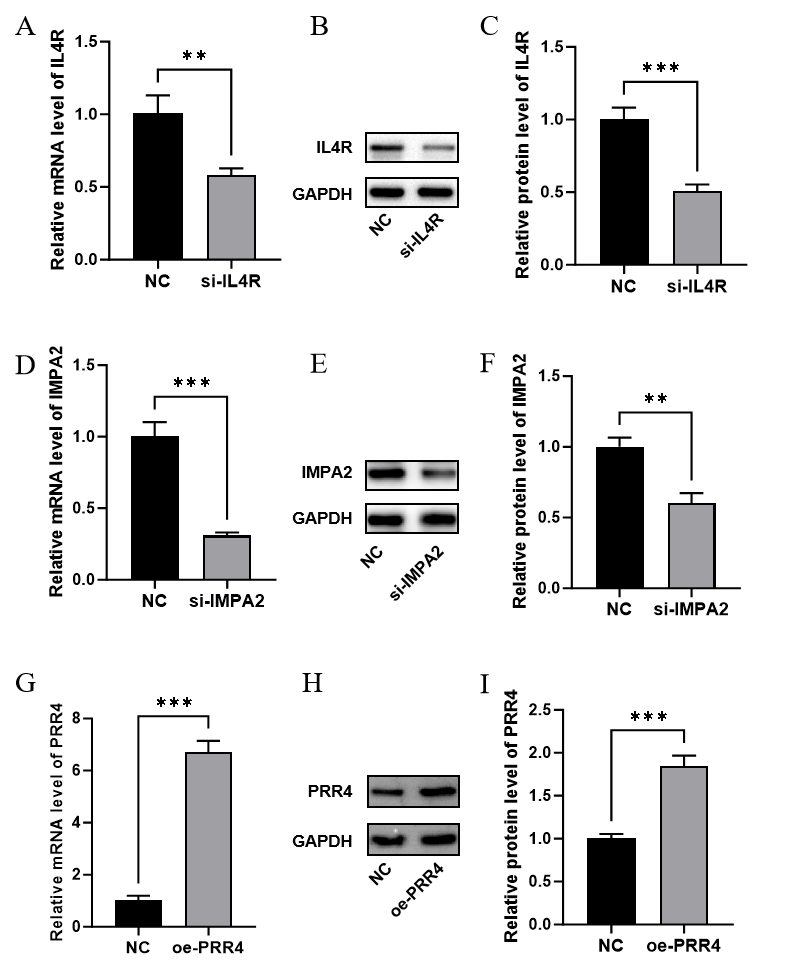


**Supplementary 5 Validation of IL4R and IMPA2 knockdown and PRR4 overexpression.**

(A–C) qRT-PCR and Western blot results showing that IL4R expression was significantly decreased in the si-IL4R group. (D–F) qRT-PCR and Western blot results showing that IMPA2 expression was significantly decreased in the si-IMPA2 group. (G–I) qRT-PCR and Western blot results showing that PRR4 expression was significantly increased in the oe-PRR4 group. ***P < 0.0001, **P < 0.01
